# miR-504 suppresses mesenchymal phenotype of glioblastoma by directly targeting the FZD7-mediated Wnt–β-catenin pathway

**DOI:** 10.1186/s13046-019-1370-1

**Published:** 2019-08-16

**Authors:** Qiang Liu, Yanlei Guan, Zhenhang Li, Yao Wang, Yu Liu, Run Cui, Yunjie Wang

**Affiliations:** 1 Department of Neurosurgery, First Affiliated Hospital of China Medical University, Shenyang, 110001 Liaoning China; 2 Department of Cardiac Surgery, First Affiliated Hospital of China Medical University, Shenyang, 110001 Liaoning China; 3 Guangdong Key Laboratory for Biomedical Measurements and Ultrasound Imaging, Laboratory of Evolutionary Theranostics, School of Biomedical Engineering, Health Science Center, Shenzhen University, Shenzhen, 518060 China

**Keywords:** GBM, miR-504, Mesenchymal phenotype, EMT, FZD7, Wnt–β-catenin pathway

## Abstract

**Background:**

MicroRNAs (miRNAs) play crucial roles in tumor initiation and development. Previously, we indicated that miR-504 is downregulated and suppresses tumor proliferation in glioblastoma (GBM). However, the regulation and relevant mechanism of miR-504 in GBM mesenchymal (ME) transition remain unclear.

**Methods:**

Transcriptome and clinical data were obtained from The Cancer Genome Atlas (TCGA) database. The potential functions of miR-504 were predicted using gene ontology analysis. GBM cell migration and invasion were examined using wound healing and Transwell assays. Epithelial–mesenchymal transition (EMT) progression in GBM cell lines was detected with immunofluorescence and western blotting. The stemness activity of glioma stem-like cells (GSCs) was assessed by sphere formation assay and tumor xenograft model. miR-504 binding to the FZD7 (frizzled class receptor 7) 3′ untranslated region (3′UTR) was validated using dual luciferase reporter assay. TOP/FOP Flash assays were conducted to determine the effects of miR-504 on Wnt/β-catenin signaling.

**Results:**

Analysis of TCGA transcriptomic data showed that low miR-504 expression correlated with ME subtype transition and poor survival in patients with GBM. Functional experiments showed that miR-504 overexpression suppressed malignant behaviors of GBM cells, such as migration, invasion, EMT, and stemness activity. Furthermore, miR-504 was a negative regulator of the Wnt–β-catenin pathway by directly repressing FZD7 expression, and FZD7 overexpression reversed the EMT inhibition caused by miR-504. Moreover, the low miR-504/FZD7 expression ratio was a ME subtype marker and could serve as a significant prognostic indicator and predict the clinical outcome of chemotherapy and radiotherapy for patients with GBM in TCGA dataset.

**Conclusions:**

Our results suggest that miR-504 suppresses the aggressive biological processes associated with the ME phenotype of GBM and could be a potential candidate for therapeutic applications in these malignant brain tumors.

**Electronic supplementary material:**

The online version of this article (10.1186/s13046-019-1370-1) contains supplementary material, which is available to authorized users.

## Background

Glioblastoma (GBM) is the most common and aggressive type of malignant tumor of the central nervous system in human adults. Despite the investment of considerable effort in improving diagnosis and treatment, it remains one of the deadliest malignancies worldwide [1]. The current treatment strategy for patients with GBM is surgical resection followed by combined chemo-radiotherapy; however, the prognosis has not seen any meaningful improvement [2]. The unfavorable efficacy is mainly due to the high proliferation, invasiveness, and therapeutic resistance of GBM. Hence, there is an urgent need to understand the molecular mechanisms involved in the malignant behaviors of GBM and to explore novel targets for GBM treatment.

In recent years, many groups have performed integrated analyses based on high-throughput genome and transcriptome profiling to comprehensively explore the molecular mechanism of GBM [3–5]. As the most representative molecular pathological subclassification scheme, integrated genomic analysis by Verhaak et al. classifies GBM into four subtypes: proneural (PN), neural (NE), classical (CL), and mesenchymal (ME). Among them, the PN and ME subtypes have been identified consistently in various classification systems and have been indicated to have different biological behaviors and distinct clinical prognoses [4, 5]. Compared with PN subtype tumors, ME subtype GBM has more aggressive phenotypes, such as radioresistance and chemoresistance, increased invasiveness, and reduced cell stiffness, leading to therapeutic failure and poor prognosis [6, 7]. On the other hand, similar to tumor cells undergoing epithelial–mesenchymal transition (EMT) to obtain more aggressive characteristics the shift towards the ME subtype appears to be a common pattern during the malignant progression of GBM [8–10]. Furthermore, revealing an aggressive phenotype, ME subtype GBM typically expresses neural stem cell markers, and EMT is an important inducer of cancer stem cell (CSC) properties [11, 12]. Taking into account the extremely aggressive behavior of ME subtype GBM, it is necessary to clarify the activating molecular mechanism and the transition of the ME phenotype in these malignant brain tumors.

Accumulating evidence has indicated that microRNAs (miRNAs) play important roles in GBM tumorigenesis by functioning as both oncogenic promoters and tumor suppressors [13–19]. These specific miRNAs can potentially serve as effective biomarkers for improving diagnostic and prognostic accuracy, or as targets for novel treatment strategies in patients with GBM. However, the mechanism of miRNA regulation of ME transition in GBM remains unclear.

Previously, we reported that miR-504 is downregulated in both tumor tissues and cell lines and correlates with poor prognosis in high-grade glioma, including GBM [14, 20]. Evaluating the relationship between miRNA and mRNA expression statistically, we found that miR-504 expression correlated negatively with the expression of ME markers in GBM [21]. Furthermore, our recent study demonstrated that miR-504 could inhibit cell proliferation and promote apoptosis by targeting FOXP1 (forkhead box P1) in glioma cells [20]. These findings suggested the possibility that miR-504 might have an important role in regulating ME phenotype transition in GBM.

In the present study, we aimed to clarify the function and mechanism of miR-504 in the biological regulation of malignant ME phenotypes and to evaluate the possibility of it being a prognostic predictor in GBM. First, The Cancer Genome Atlas (TCGA) transcriptomic data analysis showed that low miR-504 expression correlated with ME subtype transition and poor survival in patients with GBM. In addition, miR-504 suppressed malignant ME phenotypes such as migration, invasion, EMT, and stemness activity in GBM cells. Furthermore, we identified frizzled class receptor 7 (FZD7) as a target gene of miR-504, and demonstrated that miR-504 could negatively regulate the Wnt–β-catenin pathway by directly repressing FZD7 expression. Moreover, we confirmed that the miR-504/FZD7 expression ratio was a ME marker and could serve as a significant prognostic indicator and predict the clinical outcome of chemotherapy and radiotherapy for patients with GBM in TCGA dataset. Conclusively, our results suggest that miR-504 suppresses the aggressive biological processes associated with ME phenotype GBM and could be a potential candidate for therapeutic applications in these malignant brain tumors.

## Methods

### Clinical data

Processed miRNA and mRNA expression data (level 3) as well as the clinicopathological annotations were downloaded from TCGA portal (http://cancergenome.nih.gov). miRNA and mRNA expression was profiled using Affymetrix HT Human Genome U133A microarrays. Samples containing both miRNA and mRNA expression data were selected for analysis. A total of 517 GBM samples and 10 normal brain tissues (NBT) were enrolled in this study. Archived paraffin-embedded GBM tissues were collected from patients (*n* = 50) who had undergone surgery at the Department of Neurosurgery, The First Affiliated Hospital of China Medical University.

### Principal components analysis (PCA) and gene ontology (GO) analysis

Correlation analysis of miR-504 was performed in gene expression profiles available in TCGA dataset using R software (version 3.4.2), and the top 200 negatively related genes and top 200 positively related genes were collected for analysis. Meanwhile, samples were divided into miR-504 high- and low-expression groups according to the upper quartile value of miR-504 expression levels, and the top 200 significantly upregulated genes in the miR-504 low-expression group were summarized using Microsoft Excel 2010. PCA was performed using R programing language to assess the expression patterns of grouped patients. To identify the biological processes associated with miR-504 in GBM, the top 200 negatively related genes and differentially upregulated genes described above were analyzed using the DAVID (Database for Annotation, Visualization and Integrated Discovery) online tool (https://david.ncifcrf.gov/). We used the same screening criteria and grouping methods to explore the biological processes associated with the miR-504/FZD7 ratio.

### Gene set enrichment analysis (GSEA) and gene set variation analysis (GSVA)

GSEA (https://www.broadinstitute.org/) was used to explore whether the identified gene sets displayed statistical differences between two groups. The statistical significance was determined using the normalized enrichment score (NES) and false discovery rate (FDR). The association between the miR-504/FZD7 ratio and ME phenotypes was verified with GSVA. The gene sets involved in our study were downloaded from MSigDB (molecular signatures database, https://www.broadinstitute.org/gsea/msigdb/, Additional file 1: Table S1).

### Cell culture

The U87 and U373 cell lines were purchased from the Chinese Academy of Sciences cell bank. The cells were grown in Dulbecco’s modified Eagle’s medium (DMEM, HyClone) supplemented with 10% fetal bovine serum (FBS, Gibco).

### GSC isolation and culture

GSC-U87 were isolated from U87 cells using a serum-free clone formation method. Briefly, 5 × 10^5^ U87 cells were seeded in flasks and cultured in serum-free culture medium until several primary tumor spheres were visible under light microscopy. The serum-free culture medium (stem cell medium) consisted of DMEM/F12 medium (Gibco) supplemented with epidermal growth factor (EGF, 20 ng/ml, Gibco), basic fibroblast growth factor (bFGF, 20 ng/ml, Gibco), and 2% B27 (50×, Gibco). The GSC primary cell line GSC-1295 was isolated from a GBM specimen which was taken from the center of the tumor during surgery. Viable fragments were transferred to a beaker containing 0.25% trypsin in 0.1 mM EDTA (4,1) and slowly stirred at 37 °C for 1 h. Subsequently, dissociated cells were seeded in 75 cm^2^ culture flasks at 3000 cells/cm^2^ in stem cell medium. After 2 weeks in culture, the spheres were observed under light microscopy.

GSC-U87 and GSC-1295 cells were cultured in DMEM/F12 medium (Gibco) supplemented with EGF (20 ng/ml, Gibco), bFGF (20 ng/ml, Gibco), and 2% B27 (50×, Gibco). All cells were maintained at 37 °C under a humidified atmosphere containing 5% CO_2_.

### Establishment of stable cell lines

The LV-hsa-miR-504 vector was constructed using commercially available lentiviral vectors (GeneChem). After verification by DNA sequencing, the vector was used to overexpress miR-504 in U87, U373, GSC-U87, and GSC-1295 cells. LV-anti-hsa-miR-504 vector was used to knockdown miR-504 expression. The transfection was based on the manufacturer’s manual. LV-empty vectors (miR-NC and anti-NC) acted as the negative controls (NC). Green fluorescent protein (GFP) expression was observed using inverted fluorescence microscopy at 72 h post-infection. The transfected cells were screened with 10 μg/ml puromycin (Sigma). Quantitative reverse transcription–PCR (qRT-PCR) was performed to analyze miR-504 expression. Lentiviral vector containing the FZD7 coding sequence (LV-FZD7, GeneChem) was used to overexpress FZD7 in U87 and U373 cells stably expressing miR-504; the negative control was LV-NC. Then, FZD7 expression was analyzed using qRT-PCR and western blotting. Additional file 2 shows the detailed sequence of the lentivirus used in this experiment.

### Wound healing assay

Cells were seeded in a 6-well plate and incubated at 37 °C until they were at least 90% confluent. A scratch was made using a sterile 20-μl tip and washed with phosphate-buffered saline (PBS). Then, the cells were cultured for 24 h in serum-free DMEM. The width of the wound gaps were measured using National Institutes of Health (NIH) ImageJ software and normalized to the time 0 wounds in three independent experiments.

### Cell invasion assay

The cell invasion assay was designed in a 24-well invasion chamber system (BD Biosciences) with a polycarbonate membrane (diameter, 6.5 mm; pore size, 8 μm pre-coated with Matrigel (Corning). Cell suspension (1 × 10^5^ cells/ml) was prepared in serum-free medium, and 200 μl cell suspension was added to the upper chamber. Then, the lower chambers were filled with 500 μl medium containing 10% FBS. After 24-h incubation at 37 °C, the non-invasive cells on the top side of the membrane were removed by scraping. The filters were fixed in 10% formaldehyde and stained with 0.1% crystal violet for 15 min. The cells in three random light microscope fields were counted. All assays were carried out in triplicate.

### Tube formation assay

A 96-well plate was coated with 50 μl of Matrigel (Corning) and incubated at 37 °C for 30 min. Then, HUVECs (2× 10^5^ cells/ml) were cultured in 96-well plate with GBM cell supernatant that were collected from previously established stable cell lines, respectively. HUVECs were putted into incubator (37 °C, 5% CO2) and photographs were taken by phase contrast microscope after 6 h.

### RNA extraction and qRT-PCR

Total RNA of the GBM cells was extracted using TRIzol (Ambion) according to the manufacturer’s instructions. A Mir-X miRNA First-Strand Synthesis Kit (Takara) and PrimeScript RT Reagent Kit (Takara) were used to synthesize complementary DNA (cDNA) from miRNA and mRNA, respectively. Subsequently, the expression levels of miR-504, FZD7, CD44, and c-MYC were quantified by qRT-PCR using SYBR Premix Ex Taq II (Takara). U6 expression was used as an endogenous control for miR-504, while GAPDH (glyceraldehyde-3-phosphate dehydrogenase) was used as an internal control for FZD7,CD44 and c-MYC. Relative expression changes were calculated using the comparative threshold cycle (2^−ΔΔCt^) method. The primers used are shown in Additional file 3: Table S2.

### Protein isolation and western blotting

Total protein was extracted using radioimmunoprecipitation assay buffer according to the manufacturer’s protocol, and the protein concentration was determined using a bicinchoninic acid protein assay kit (Beyotime Biotec). Protein samples were fractionated using 8–15% sodium dodecyl sulfate–polyacrylamide gel electrophoresis and transferred to polyvinylidene fluoride membranes (Millipore). The membranes were blocked with 5% non-fat milk in TBS-T and incubated overnight at 4 °C with the following antibodies: FZD7 (Abcam); E-cadherin, N-cadherin, vimentin, SNAI2 (snail family transcriptional repressor 2), CD44, CD133 (prominin 1), nestin, SOX2 (SRY-box 2), GSK3β (glycogen synthase kinase 3 beta), β-catenin, c-MYC (Proteintech), and phosphorylated p-GSK3β (Ser9) (Cell Signaling Technology); the endogenous control was GAPDH (Proteintech), and the nuclear control was histone H3 (Abcam). The immune complexes were detected using enhanced chemiluminescence.

### Immunofluorescence

Immunofluorescence staining was performed following a standard protocol. Briefly, adhered cells were fixed with 4% paraformaldehyde (PFA) for 30 min at room temperature. Subsequently, the samples were permeabilized in 0.5% Triton X-100 and blocked in 5% blocking buffer for 1 h. The cells were then incubated at 4 °C overnight with specific primary antibody (anti-vimentin, anti-FZD7). After washing with PBS three times, the cells were incubated with fluorescence-conjugated secondary antibody (Proteintech) for 1 h and stained with DAPI (4′ 6-diamidino-2-phenylindole) for 10 min, and viewed under a fluorescence microscope.

For GSC identification, tumor spheres were placed on poly-L-ornithine (BD Biosciences)-coated glass coverslips, incubated with anti-CD133 (1:100, Proteintech), and stained with Cy3-conjugated secondary antibody (Proteintech). For the differentiation assay, tumor spheres were seeded in a 24-well plate and cultured in medium supplemented with 10% FBS for 5 days. Differentiated cells were incubated with anti-GFAP (glial fibrillary acidic protein) antibody (1:100, Proteintech), stained with Cy3-conjugated secondary antibody (Proteintech), and counterstained with DAPI.

### Spheroid formation assay

We seeded 500 cells in 6-well ultra-low cluster plates and cultured them in DMEM/F12 medium (20 ng/ml EGF, 20 ng/ml FGF, 2% B27). After 2 weeks, tumor spheres with diameter > 70 μm were manually counted in every well. Sphere formation efficiency was calculated as the percentage of seeded cells that gave rise to a spheroid.

### Tumor xenograft model

All animal procedures were performed in accordance with the NIH Guide for the Care and Use of Laboratory Animals. For the subcutaneous transplantation experiment, 5–6-week-old female immune-deficient nude mice (BALB/c null) were purchased from Beijing Vital River Laboratory Animal Technology Co., Ltd. The mice were housed and maintained in laminar flow cabinets under specific pathogen–free conditions and were fed ad libitum. GSC-U87 cells (1 × 10^6^) in 0.2 ml PBS were injected into the back flanks of the mice. Tumor sizes were measured using Vernier calipers. The tumor volume (V, mm^3^) was determined using the following formula: (L × W^2^)/2, where L is the tumor length and W is the tumor width. The mice were sacrificed 6 weeks after implantation, and the tumors were weighed and photographed. To establish intracranial gliomas, U87MG cells (2.5 × 10^5^) were infected with miR-504 or miR-NC virus and then implanted stereotactically into the frontal lobe of nude mice (5 mice for each group). The mice were observed daily for any possible signs of distress and the survival rate was recorded from the time of injection.

### Immunohistochemistry

Sections (4 μm) were obtained from PFA-fixed, paraffin-embedded tissues of human GBM tissue and xenograft tissue. The sections were dewaxed and washed with ethanol. Then, the slides were blocked with 10% normal goat serum and incubated with primary antibody (anti-FZD7, anti-CD133, anti-KLF4, anti-vimentin, anti-β-catenin) at 4 °C overnight. After 1-h hybridization with secondary antibody, the sections were incubated with VECTASTAIN Elite avidin–biotin complex reagent (Vector Laboratories) for 15 min. The subsequent assays were performed according to the manufacturer’s protocols for the diaminobenzidine (DAB) kit (BD Biosciences) and the sections were stained with hematoxylin. Images were obtained using a BX-51 light microscope (Olympus).

### Dual luciferase reporter assay

The dual luciferase reporter assay was conducted to confirm whether FZD7 was the target gene of miR-504. HEK-293 T cells were plated in 96-well plates at a density of 2 × 10^4^ cells per well before transfection. After 24-h culture, the cells were transfected with pMIR-REPORT-FZD7–3’UTR-mut, pMIR-REPORT-FZD7–3’UTR-wt plasmid (OBIO), and miR-504 mimics (GenePharma). After 6-h incubation, firefly and Renilla luciferase activity was measured using a Dual-Luciferase Reporter Assay System (Promega). Renilla luciferase activity was used as the internal control.

### TOP/FOPFlash reporter assay

TOP/FOPFlash reporter assays were used to determine the transcriptional activity of the Wnt–β-catenin pathway. The reporter plasmids containing TOPFlash or mutated FOPFlash TCF/LEF DNA binding sites were purchased from Addgene. Briefly, cells were seeded in a 96-well plate and transfected with miR-504 mimics or inhibitor (GenePharma) in the presence of the TOPFlash or FOPFlash vector and pRL-TK vector (Promega). Luciferase activity was measured using the Promega Dual-Luciferase Reporter Assay System 24 h after transfection. The luciferase activity of each sample was normalized with the respective Renilla luciferase activity.

### Statistical analysis

Statistical analyses were performed using Microsoft Excel 2010, GraphPad Prism 5, SPSS 19, and R software. The difference between two groups was calculated using Student’s t-test. The linear relationship between the expression levels of different genes was evaluated using the Pearson correlation. The association between the miR-504/FZD7 ratio and GBM molecular subtype was evaluated using the chi-square test. Survival distributions were estimated using Kaplan–Meier survival analysis, and statistical significance was assessed using the log-rank test. Independent prognostic factors were identified using univariate and multivariate Cox regression analyses. All experiments were performed in triplicate and the data are expressed as the mean ± standard deviation (SD). Differences were considered significant at *P* < 0.05.

## Results

### miR-504 downregulation correlated with ME subtype and poor survival in GBM

**Fig. 1:**
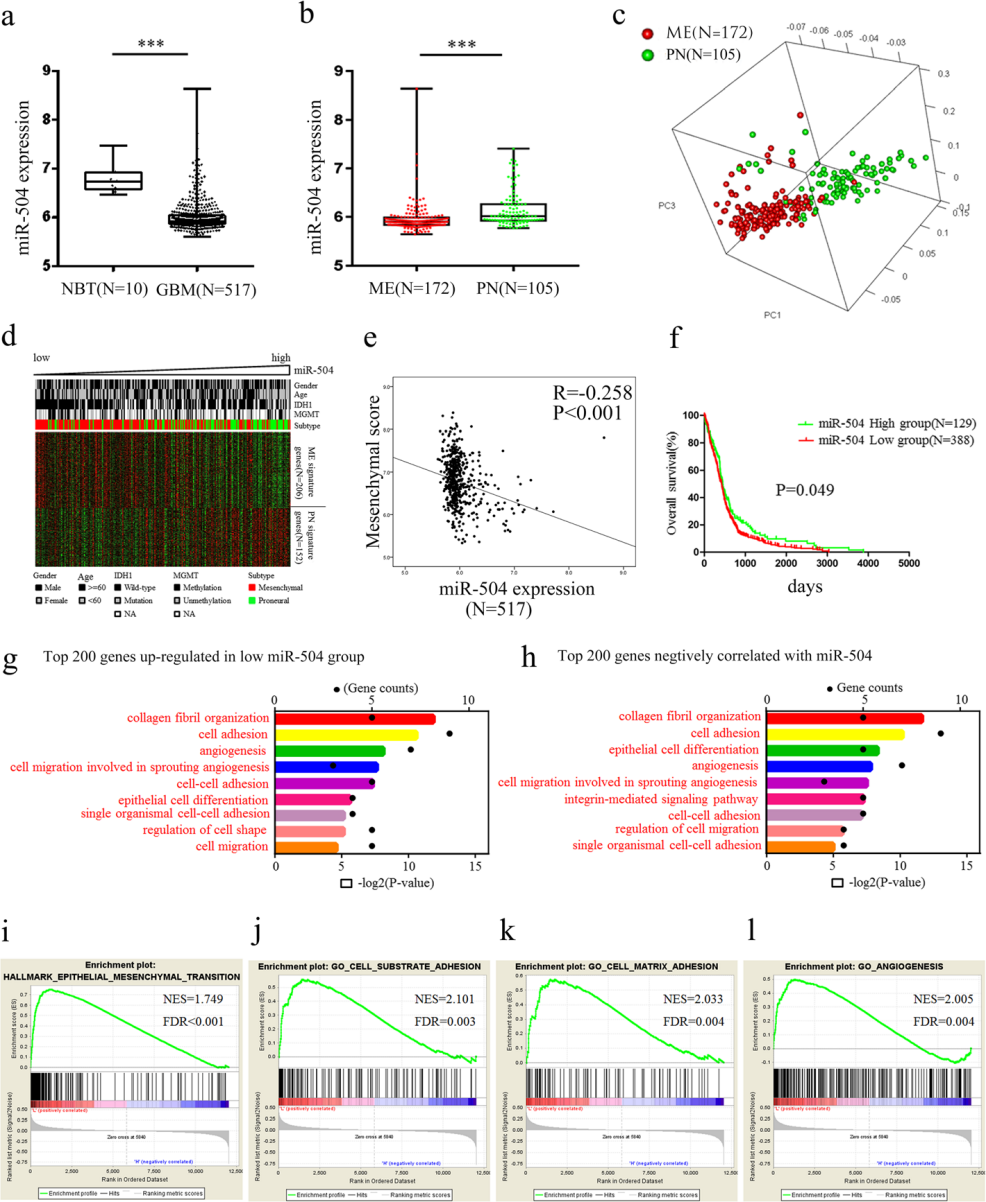
Downregulation of miR-504 in ME subtype GBM. **a** Analysis of TCGA data of miR-504 expression levels in NBT (*n* = 10) and GBM (*n* = 517). **b** Analysis of TCGA data of miR-504 expression levels in ME subtype GBM (*n* = 172) and PN subtype GBM (*n* = 105). **c** PCA based on the miR-504–related genes shows a distinct distribution pattern between patients with the ME and the PN subtype. **d** The expression pattern of ME and PN signature genes corresponding to miR-504 expression. **e** Pearson correlation analysis between miR-504 and ME score in TCGA. **f** Kaplan–Meier analysis of overall survival of patients with GBM from TCGA database according to miR-504 expression level. **g** GO analysis of the top 200 differentially upregulated genes in miR-504 low-expression tumors. **h** GO analysis of top 200 genes negatively related to miR-504. **i**–**l** GSEA highlighting positive association of decreased miR-504 expression levels with EMT, cell adhesion, and angiogenesis. ***P < 0.001

ME subtype GBM has more aggressive phenotypes such as poor prognosis and radioresistance and chemoresistance as compared with PN tumors. To reveal the relationship between miR-504 and the malignant ME phenotype in GBM, we analyzed transcriptomic data, including the expression of both miR-504 and mRNAs in 517 GBM samples (including 172 ME tumors and 105 PN tumors) and 10 NBT in TCGA dataset. miR-504 expression was markedly decreased in GBM tissues compared with that in NBT (*P* < 0.001, Fig. 1a). Patients with ME subtype GBM had significantly low levels of miR-504 expression than patients with PN subtype GBM (*P* < 0.001, Fig. 1b). Subsequently, we used a Pearson correlation test to select genes (detailed in Additional file 4: Table S3) that showed a significant correlation with miR-504. PCA based on the miR-504–related genes showed a distinct distribution pattern between patients with the ME and PN subtypes, indicating different expression of miR-504 and its related genes among the two subtypes (Fig. 1c). In addition, the ME and PN signature genes exhibited distinct, different expression patterns corresponding to miR-504 expression (Fig. 1d), and miR-504 expression correlated negatively with the ME score based on the mean expression level of 206 ME signature genes (R = − 0.258, P < 0.001, Fig. 1e). Further, the patients were divided into two groups according to miR-504 expression level; Kaplan–Meier survival analysis showed that patients with miR-504 low expression had significantly shorter overall survival than those with miR-504 high expression (*P* = 0.049, Fig. 1f). We performed survival analyses on different GBM subtypes, but significant differences in survival were not observed (Additional file 5: Figure S1). Taken together, these results indicate that miR-504 downregulation correlates with the ME subtype in GBM.

### miR-504 is involved in ME phenotype–associated processes

To reveal the potential biological functions of miR-504, we explored the miR-504–associated biological processes in TCGA dataset. GO analysis was performed based on genes that were differentially upregulated in miR-504 low-expression tumors (Fig. 1g; detailed gene information is in Additional file 6: Table S4), and the genes correlated negatively with miR-504 (Fig. 1h; detailed gene information is in Additional file 4: Table S3), respectively. Our results show that the ME phenotype–associated biological processes, including cell adhesion, angiogenesis, and cell migration, were significantly enriched (Fig. 1g, h). Consistent with this, GSEA showed enrichment of ME phenotypes in GBM with low miR-504 compared to that with high miR-504 (Fig. 1i), indicating the involvement of miR-504 in ME transition–related processes in GBM.

### miR-504 inhibited ME properties and EMT in GBM cells

**Fig. 2:**
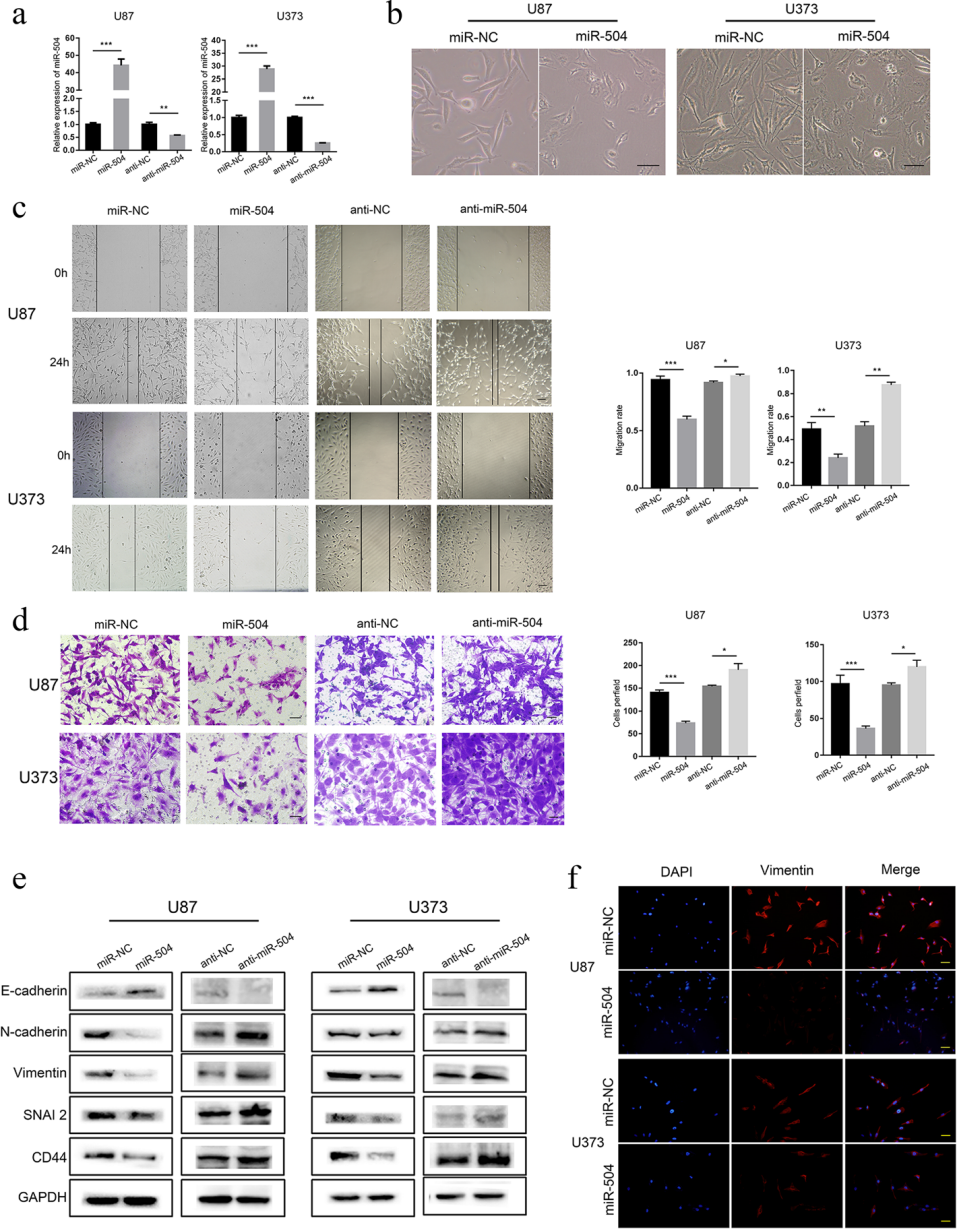
miR-504 suppresses the ME properties and EMT of GBM cells. **a** qRT-PCR detection of miR-504 expression in U87 and U373 cells transfected with miR-504 overexpression and inhibition vectors. **a** Representative images of GBM cell morphological changes after miR-504 transfection (scale bars = 100 μm). (c, d) Representative results of wound healing assays (**c**) and Transwell assays (**d**) showing the effect of miR-504 overexpression and inhibition on the migration and invasive abilities of GBM cells (scale bars = 100 μm). **e** Western blot analysis of the levels of representative epithelial and ME markers. **f** Immunofluorescence examination of ME marker expression in U87 and U373 cells transfected with miR-504 or miR-NC (red, vimentin; blue, DAPI; scale bars = 100 μm). Data are the mean ± SD of three independent experiments. *P < 0.05,**P < 0.01, ***P < 0.001

To confirm the bioinformatics functional analysis results, we investigated the role of miR-504 in the ME properties and EMT in GBM cells. We first constructed miR-504 stable overexpression and knockdown GBM cell lines (U87 and U373, respectively) by lentivirus vector transduction (Fig. 2a). Based on ectopic miR-504 expression, tumor cells displayed a remarkable morphological change from the typical spindle-like shape to an epithelial-like shape (Fig. 2b). Subsequently, the wound healing and Transwell invasion assays showed that exogenous miR-504 overexpression significantly suppressed GBM cell migration and invasion, respectively, while miR-504 knockdown had the opposite effect (Fig. 2c, d). Similarly, the tube formation assay showed that the number of branches were decreased in the miR-504 overexpression group, and were increased in the miR-504 inhibition group (Additional file 7: Figure S2). We also measured the expression levels of the epithelial marker E-cadherin and the ME markers N-cadherin, vimentin, SNAI2, and CD44 in transfected U87 and U373 cells to determine whether miR-504 regulates EMT in GBM cells. Western blotting revealed that miR-504 overexpression decreased N-cadherin, vimentin, SNAI2, and CD44 expression remarkably in both the U87 and U373 cells (Fig. 2e). On the contrary, E-cadherin was significantly upregulated compared with the controls. However, in the miR-504 knockdown group, N-cadherin, vimentin, SNAI2, and CD44 were upregulated, while E-cadherin was downregulated. The immunofluorescence assay confirmed this observation, showing that cells with miR-504 overexpression had weaker vimentin staining than the negative controls (Fig. 2f). To further explore the function of miR-504 in suppressing the mesenchymal properties, orthotopic xenografts were established by implanting U87 cells tanfected with miR-504 or miR-NC in nude mice. We found that overexpression of miR-504 resulted in more circumscribed borders (Additional file 8: Figure S3a), significantly smaller tumor volumes and the median survival time was longer than that of the control group (Additional file 8: Figure S3b-c). Moreover, immunohistochemistry showed that the protein level of mesenchymal classical makers, β-catenin and Vimentin, were decreased in exnografts of miR-504 group (Additional file 8: Figure S3d). Taken together, miR-504 overexpression suppresses the ME properties and EMT in GBM cells.

### miR-504 attenuated the stemness activity of GSCs

**Fig. 3:**
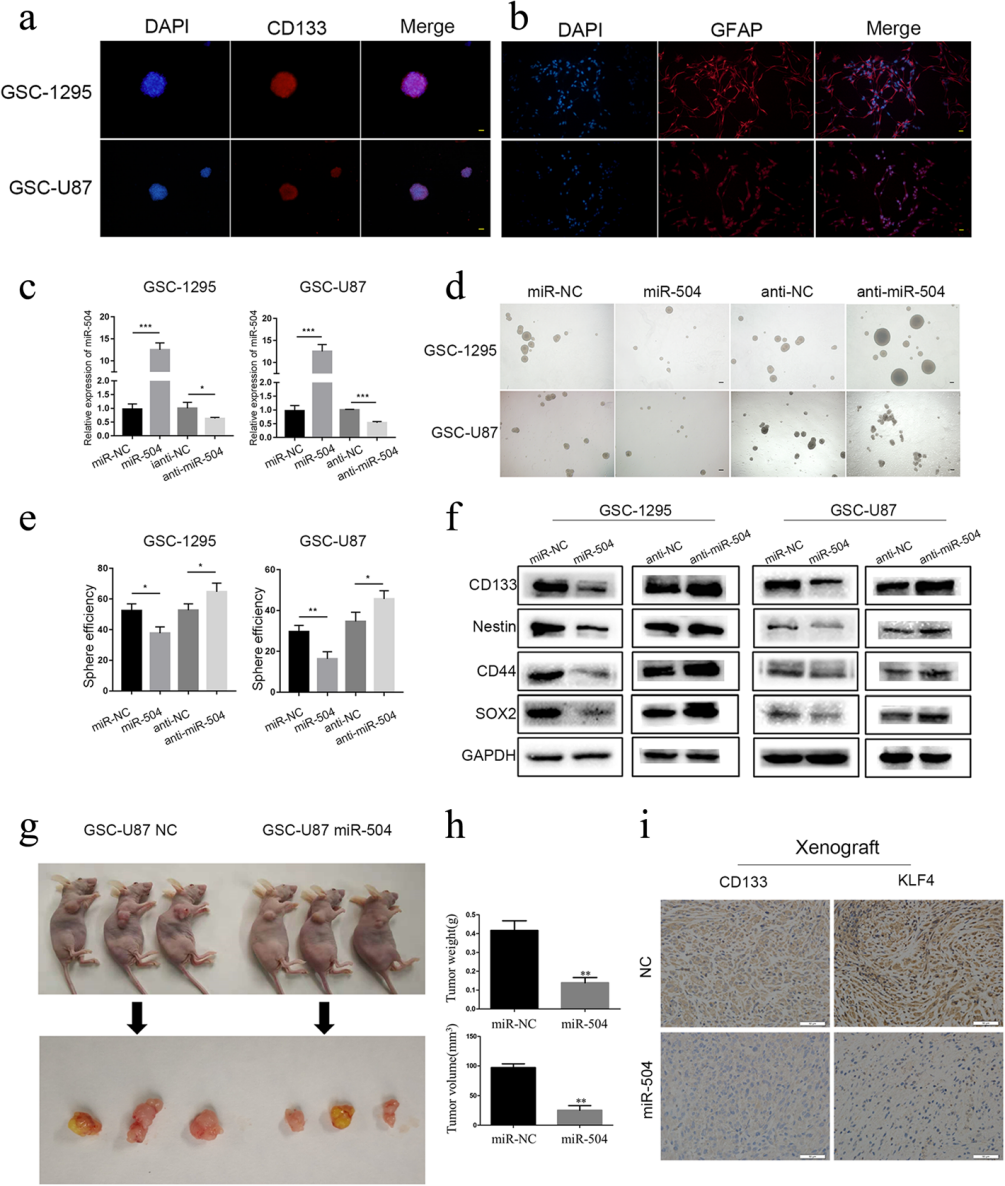
miR-504 attenuates GSC stemness activity. **a** Immunofluorescence showing tumor spheres comprised of CD133-positive GSCs (red, CD133; blue, DAPI; scale bars = 100 μm). **b** GSCs differentiated into GFAP-positive cells after serum induction (red, GFAP; blue, DAPI; scale bars = 100 μm). **c** qRT-PCR detection of miR-504 expression in GSC-U87 and GSC-1295 cells transfected with miR-504 overexpression and inhibition vectors. **d** Representative images of tumor spheres formed after 14-days culture (scale bars = 500 μm). **e** Western blot analysis of the levels of the representative stem cell markers. **f**–**h** miR-504 reduced GSC-U87 cell tumorigenicity in vivo. **i** Immunohistochemistry for CD133 and KLF4 in xenograft sections (scale bar = 50 μm). Data are the mean ± SD of three independent experiments. *P < 0.05, **P < 0.01, ***P < 0.001

Accumulating evidence has indicated that GSCs are sources of oncogenesis, recurrence, invasion, and chemoresistance in GBM [22–24]. On the other hand, it has been proposed that the EMT program is closely associated with the presence and activity of CSCs [25]. We therefore detected the modulation effect of miR-504 on the stemness activity of GSCs using GSC-U87 cells and primary GSC spheres, GSC-1295. We first identified GSCs in GSC-U87 and GSC-1295 cells using immunofluorescence staining. The positive staining of the neural stem cell lineage marker CD133 demonstrated that it was expressed on the membranes of most cells within the spheres (Fig. 3a). The differentiation potential of these spheres was confirmed by the high expression of GFAP following culture in DMEM containing 10% FBS without EGF or FGF (Fig. 3b). Subsequently, miR-504 stable overexpression and knockdown GSC cell lines were constructed by lentivirus vector transfection (Fig. 3c). The sphere-forming assay was used to determine whether miR-504 regulates the self-renewal ability of GSCs, and the results indicated that overexpression of miR-504 in GSCs suppressed their tumor sphere formation capability, while the inhibition of miR-504 increased it (Fig. 3d, e). In addition, western blotting showed that both GSC-U87 and GSC-1295 cells with miR-504 overexpression had significantly decreased expression of the widely identified neural stem cell markers: CD133, nestin, SOX2, and CD44, while their expression was upregulated in the miR-504 inhibition group (Fig. 3f). After demonstrating that miR-504 could inhibit the self-renewal capability and suppress the expression of neural stem cell markers in GSCs, we investigated whether this tumor-suppressive miRNA would have a similar effect in vivo, using a nude mouse xenograft model. GSC-U87 cells stably transfected with miR-504 or miR-NC were injected into the flanks of the nude mice. At 6 weeks post-inoculation, mice with GSC-87-miR-504 tumors had smaller tumors and lower tumor weight (Fig. 3g-h). In addition, immunohistochemistry confirmed reduced protein levels of the neural stem cell markers CD133 and KLF4 in xenografts generated from GSC-U87-miR-504 cells (Fig. 3i). These results demonstrate that miR-504 can suppress the tumorigenic capacity of GSCs.

### FZD7 was identified as a direct target of miR-504 in GBM

**Fig. 4:**
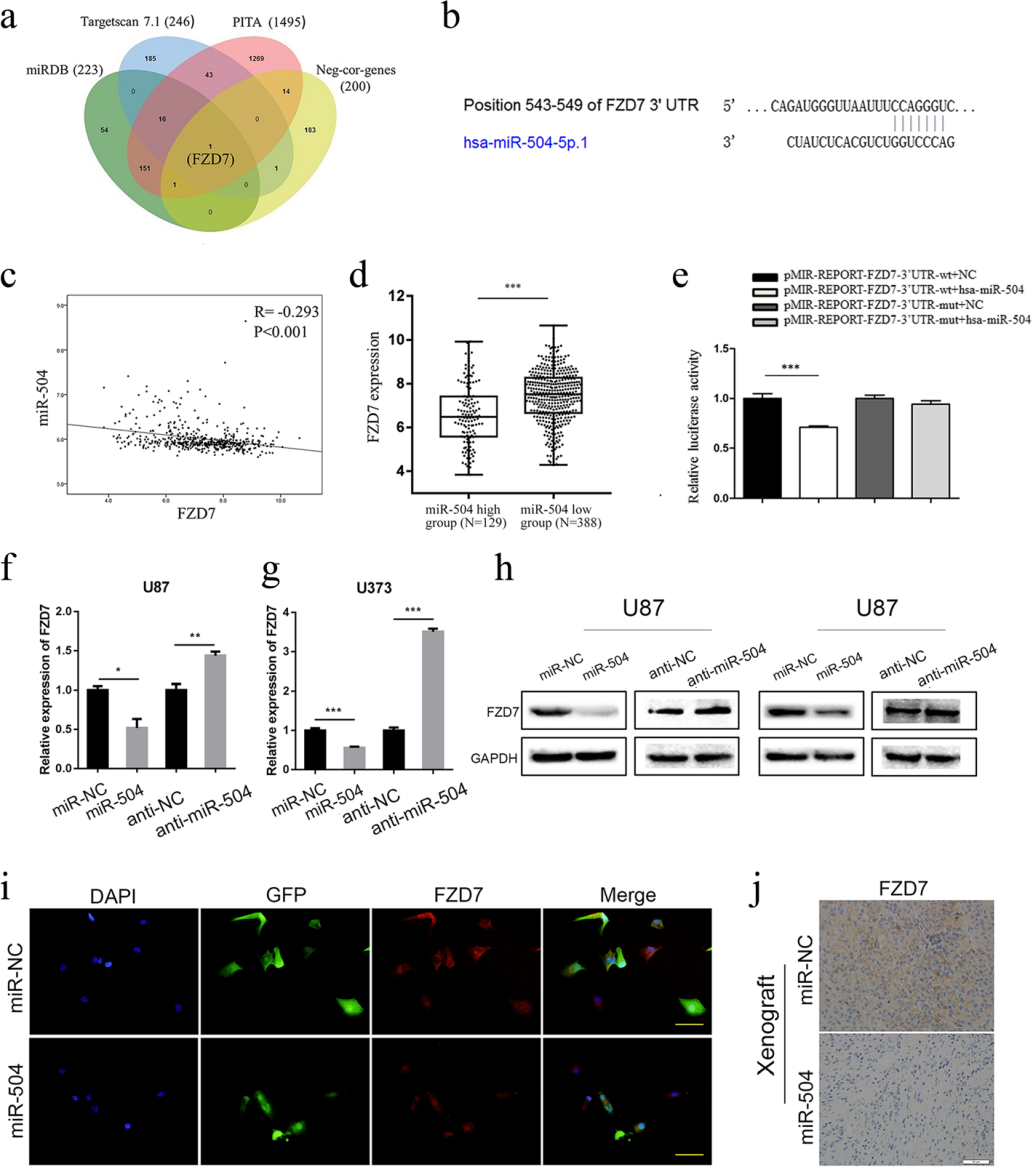
miR-504 targets the FZD7 3′UTR directly. **a** Venn diagram identifying the overlap between predicted genes and miR-504–negatively related genes. **b** The predicted target site of FZD7 3′UTR binding to miR-504. **c** Pearson correlation analysis of miR-504 and FZD7 in TCGA. **d** TCGA data analysis of FZD7 expression levels between miR-504 high-expression tumors (*n* = 129) and miR-504 low-expression tumors (*n* = 388). **e** Measurement of firefly/Renilla luciferase activity in HEK-293 T cells co-transfected with wild-type-FZD7–3′-UTR or mutant-type-FZD7–3′-UTR reporters and NC mimic or miR-504 mimic. **f**, **g** qRT-PCR determination of FZD7 mRNA levels in U87 and U373 cells after transfection with miR-504, miR-NC, anti-NC, or anti-miR-504. **h** Western blot analysis of FZD7 protein levels in U87 and U373 cells after transfection with miR-504, miR-NC, anti-NC or anti-miR-504 . **i** Immunofluorescence examination of FZD7 expression in U87 cells transfected with miR-504 or miR-NC (red, FZD7; green, GFP; blue, DAPI; scale bars = 100 μm). j Immunohistochemistry of FZD7 in xenograft sections (scale bar = 50 μm). Data are the mean ± SD of three independent experiments. *P < 0.05, **P < 0.01, ***P < 0.001

We attempted to identify the direct target genes of miR-504 to clarify the underlying mechanism of its negative regulation of the malignant phenotypes GBM. First, we predicted putative targets of miR-504 using three publicly available online algorithms (TargetScan, miRDB, PITA) and identified common candidate genes they predicted (detailed gene information is in Additional file 9: Table S5). Meanwhile, 200 genes that showed a significantly negative correlation with miR-504 were selected by the Pearson correlation test based on TCGA data (detailed gene information is in Additional file 4: Table S3). By overlapping these two gene sets, FZD7 was identified as the only candidate target gene of miR-504 (Fig. 4a). Figure 4b shows the putative binding site of miR-504 in the 3′ untranslated region (3′UTR) of FZD7 mRNA. Subsequently, the relationship between FZD7 expression and miR-504 was statistically evaluated. FZD7 showed a significantly negative correlation with miR-504 (Fig. 4c, Pearson correlation test, *P* < 0.001), and high FZD7 expression was closely associated with low miR-504 expression (Fig. 4d, *P* < 0.001). Previously, we examined the miR-504 expression level in 50 GBM samples [20]. Then, we measured FZD7 expression in the same cohort using immunohistochemistry. The samples were divided into the high expression group (*n* = 26) and low expression group (*n* = 24) according to FZD7 expression. In the FZD7 high expression group, 19 of 26 samples showed low miR-504 levels. These results indicate a significant negative correlation between miR-504 and FZD7 expression level in GBM tissues (Additional file 10: Figure S4).

We therefore performed a luciferase reporter assay to confirm the direct binding of miR-504 in the 3′UTR of FZD7 mRNA. Luciferase reporter plasmids encoding the wild-type (wt) or mutant (mut) 3′UTR region of FZD7 mRNA were constructed, and miR-504 or miR-NC was co-transfected along with the reporter plasmids into HEK-293 T cells. The miR-504/FZD7–3’UTR-wt group had significantly lower luciferase activity than the miR-NC/FZD7–3’UTR-wt group. On the contrary, the miR-504/FZD7–3’UTR-mut group and miR-NC/FZD7–3’UTR-mut group did not have significantly different luciferase activity (Fig. 4e). Furthermore, qRT-PCR and western blot demonstrated the negative regulation of FZD7 by miR-504, showing that both FZD7 mRNA and protein expression levels were remarkably decreased following miR-504 transfection and that the inhibition of miR-504 markedly promoted FZD7 expression in U87 and U373 cells (Fig. 4f–h). We performed immunofluorescence assays on U87 cells and confirmed the negative regulation of FZD7 protein by miR-504 (Fig. 4i). Moreover, immunohistochemical staining of miR-504 overexpression GSC-U87 xenograft tumors showed similar downregulation of FZD7 protein (Fig. 4j). Collectively, these results support the premise that miR-504 directly targets and negatively regulates FZD7 in GBM.

### miR-504 regulated the activation of Wnt-β-catenin signaling

**Fig. 5:**
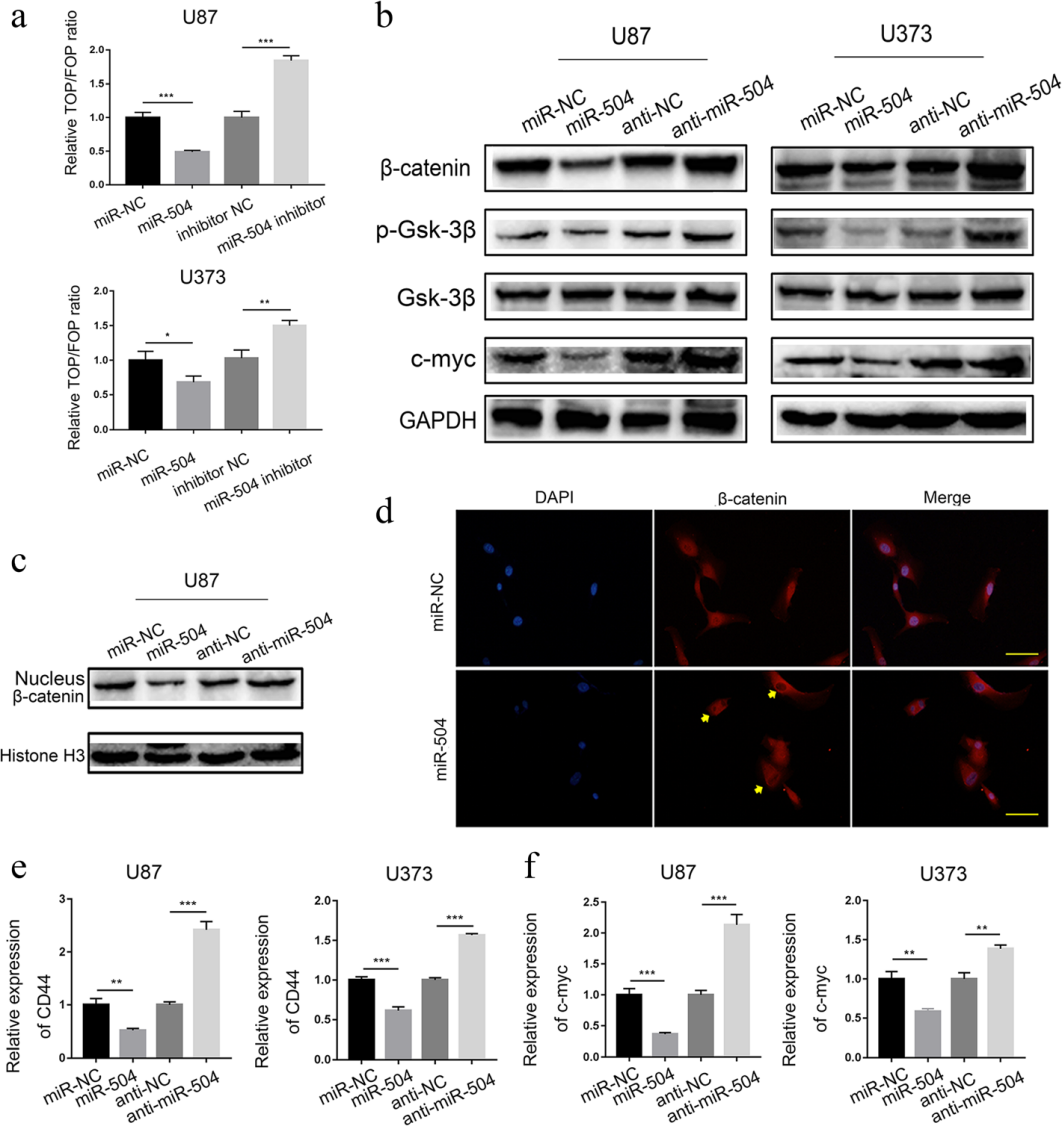
miR-504 regulates Wnt–β-catenin signaling activation. **a** TOP/FOPFlash reporter activity assay detection of relative Wnt signaling activity in U87 and U373 cells. **b** Western blot assessment of the effects of miR-504 on β-catenin, p-GSK3β, GSK3β, and c-MYC protein levels in U87 and U373 cells. **c**, **d** Western blot (**c**) and immunofluorescence (**d**) assay detection of nuclear accumulation of β-catenin in U87 cells. **e**, **f** qRT-PCR analysis of the relative mRNA expression of downstream target genes of the Wnt–β-catenin pathway, i.e., CD44 (**e**) and c-MYC (**f**), in U87 and U373 cells. Data are the mean ± SD of three independent experiments. *P < 0.05, **P < 0.01, ***P < 0.001

FZD7 regulates tumor tumorigenesis by promoting cellular β-catenin accumulation and activation of Wnt signaling, including GBM [26, 27]. Considering the function of miR-504 in regulating FZD7, we hypothesized that miR-504 may also regulate the Wnt signaling pathway. To verify our hypothesis, we first used TOP/FOPFlash reporter assays to detect the effect of miR-504 on Wnt–β-catenin signaling pathway activity, and found that it was significantly reduced in the miR-504 overexpression group, whereas the miR-504 inhibitor promoted it significantly (Fig. 5a). Then, we used western blotting to examine the expression of β-catenin, p-GSK3β, and GSK3β, important Wnt–β-catenin pathway components, in GBM cell lines. The results suggested that miR-504 overexpression markedly decreased β-catenin and p-GSK3β expression in the U87 and U373 cells, and that miR-504 inhibition distinctly increased it; GSK3β expression was not significantly changed (Fig. 5b). We also found that miR-504 overexpression decreased the nuclear translocation of β-catenin remarkably, while miR-504 inhibition increased it (Fig. 5c). In line with the above finding, immunofluorescence staining of U87 cells showed that ectopic miR-504 attenuated nuclear accumulation of β-catenin remarkably (Fig. 5d). In addition, miR-504 had a similar effect on the expression of CD44 and c-MYC, two downstream target genes of the Wnt–β-catenin pathway (Fig. 5e, f). These results all indicate that miR-504 regulates the activation of Wnt–β-catenin signaling.

### miR-504 attenuated ME signatures by suppressing the FZD7-mediated Wnt–β-catenin pathway

**Fig. 6:**
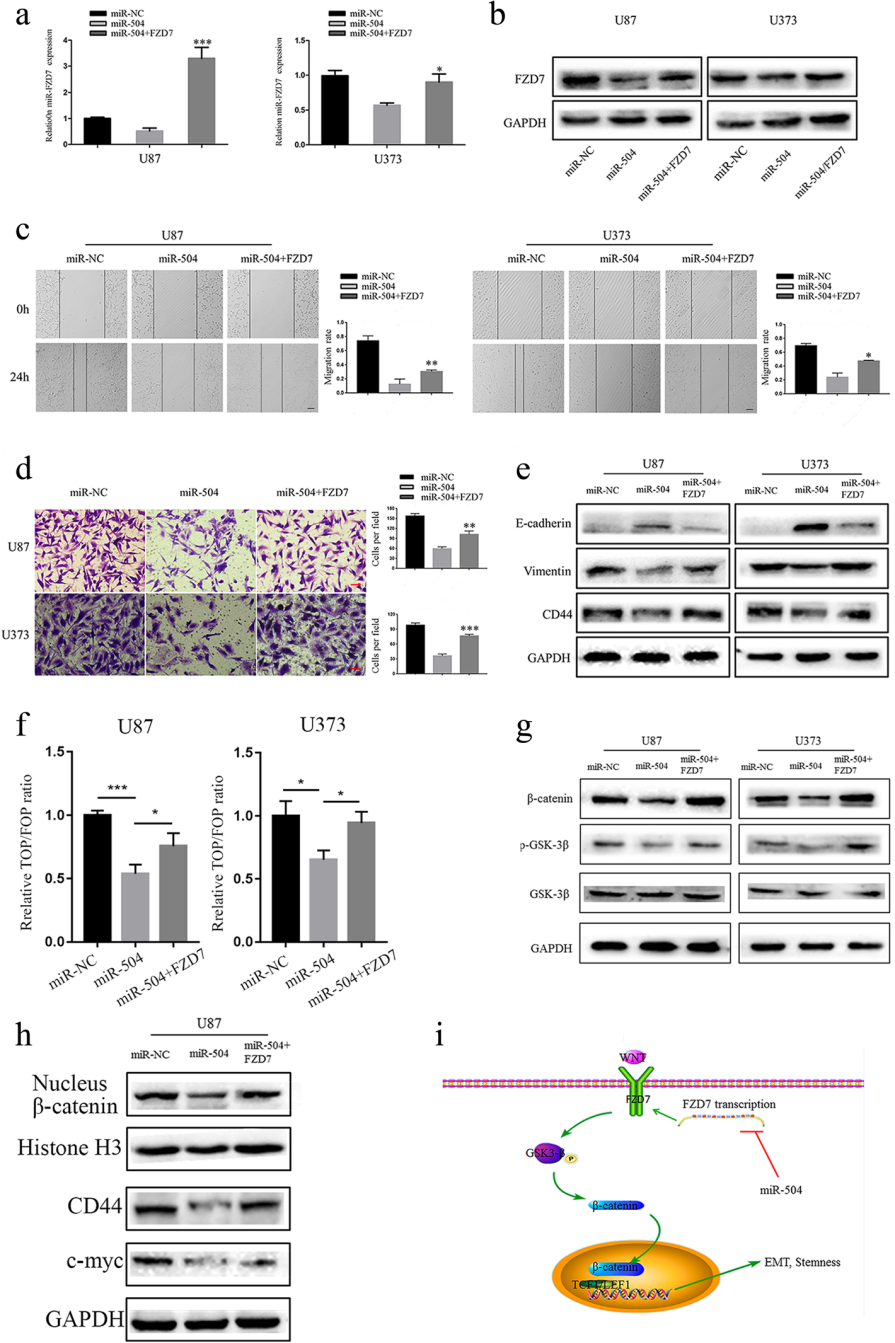
miR-504 inhibits GBM cell ME transition by suppressing the FZD7-mediated Wnt–β-catenin pathway. **a**, **b** qRT-PCR (**a**) and western blot (**b**) of FZD7 expression in miR-504–transfected U87 and U373 cells after FZD7 transfection. **c**, **d** Wound healing assay (**c**) and Transwell assay (**d**) evaluation of the restorative effect of FZD7 on miR-504–induced inhibition of migration and invasion in U87 and U373 cells (scale bar = 100 μm). **e** Western blot detection of EMT markers in U87 and U373 cells co-transfected with miR-504 and FZD7. **f** TOP/FOPFlash reporter activity detection of relative Wnt signaling activity in U87 and U373 cells. **g** Western blot detection of β-catenin, p-GSK3β, and GSK3β expression levels in miR-504 and FZD7 co-transfected cells. **h** Western blot assessment of the effects of FZD7 on nuclear β-catenin, c-MYC, and CD44 protein levels in U87 cells. **i** The diagram illustrated that miR-504 decreased the EMT and stemness via inhibiting the FZD7 mediated Wnt/β-catenin signaling pathway activity. Data are the mean ± SD of three independent experiments. *P < 0.05, **P < 0.01, ***P < 0.001

As demonstrated above, miR-504 suppressed the ME behaviors as well as EMT and Wnt/β-catenin pathway activity in GBM. We therefore used rescue experiments to investigate the functional involvement of FZD7 in these regulatory effects. First, U87 and U373 cells were co-transfected with miR-504 and FZD7 by lentivirus vectors, and qRT-PCR (Fig. 6a) and western blotting (Fig. 6b) validated the transfection efficiency. Subsequently, wound healing and Transwell invasion assays of the treated tumor cells showed that FZD7 overexpression partially reversed the inhibition of U87 and U373 cell migration (Fig. 6c) and invasion (Fig. 6d) caused by miR-504. Consistently, FZD7 overexpression also reversed the E-cadherin upregulation and vimentin and CD44 downregulation by miR-504 (Fig. 6e). Our results also demonstrate that restoring FZD7 reversed the inhibitory effect of miR-504 on Wnt–β-catenin signaling activation (Fig. 6f). Western blotting demonstrated that FZD7 overexpression could preserve p-GSK3β and β-catenin expression, as well as the β-catenin nuclear translocation (Fig. 6g, h). Overall, our results suggest that miR-504 modulates the ME signatures of GBM by downregulating the FZD7-mediated Wnt–β-catenin signaling pathway (Fig. 6i).

### Identification of the miR-504/FZD7 ratio as a novel marker of ME subtype GBM

**Fig. 7:**
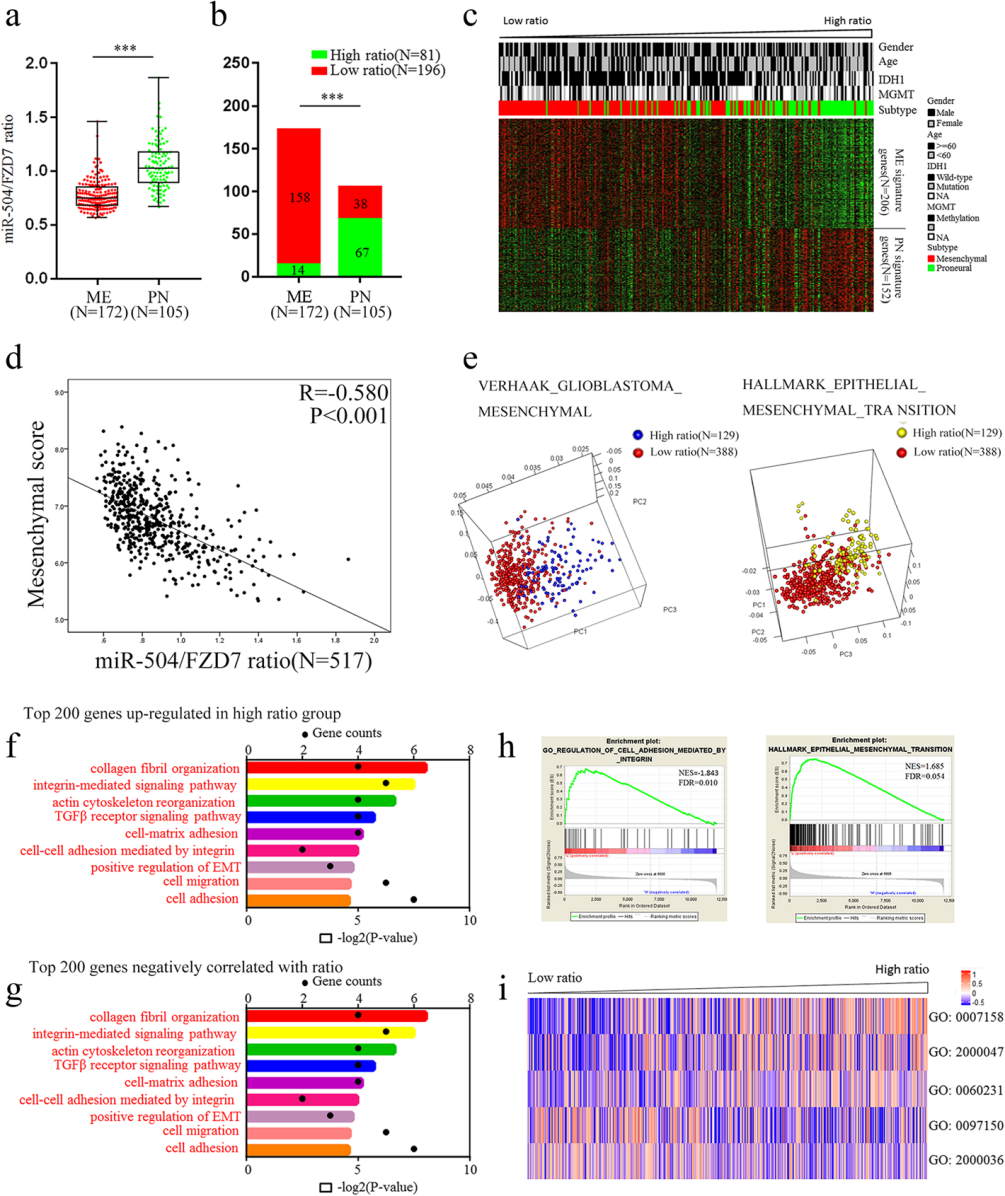
Identification of the miR-504/FZD7 ratio as a ME subtype marker in GBM. **a** Distribution of the miR-504/FZD7 ratio between ME subtype GBM (*n* = 172) and PN subtype GBM (*n* = 105) in TCGA. **b** Chi-square analysis of the relationship between the miR-504/FZD7 ratio and molecular subtype. **c** The expression patterns of ME and PN signature genes corresponding to the miR-504/FZD7 ratio. **d** Pearson correlation analysis of the miR-504/FZD7 ratio and ME score in TCGA. **e** PCA based on ME signature genes and EMT signature genes showed distinct distribution patterns between GBM of high- and low-ratio groups. **f** GO analysis of top 200 genes negatively related to the miR-504/FZD7 ratio. **g** GO analysis of top 200 differentially upregulated genes in low–miR-504/FZD7 ratio tumors in TCGA. **h** GSEA comparison of ME phenotype between high- and low–miR-504/FZD7 ratio cases. **i** Clustering of GSVA scores and analyses of biological processes associated with the miR-504/FZD7 ratio in GBM according to TCGA dataset (GO:0007158, neuron cell–cell adhesion; GO:2000047, regulation of cell–cell adhesion mediated by cadherin; GO:0060231, mesenchymal to epithelial transition; GO:0097150, neuronal stem cell population maintenance; GO:2000036, regulation of stem cell population maintenance). ***P < 0.001

After demonstrating that miR-504 could reverse ME signatures by directly targeting FZD7 in GBM, we comprehensively evaluated the potential of the combination of miR-504 and FZD7 as a novel biomarker of ME subtype GBM. First, we integrated the two molecules into a ratio (i.e., the miR-504/FZD7 ratio) based on their expression levels and explored the association between the transcriptomic features and the ratio in TCGA cohort using PCA. We found that tumors with high or low ratios tended to distribute in different directions (Additional file 11: Figure S5a), indicating a tight association between whole-transcriptome expression profiles and the miR-504/FZD7 ratio. Subsequently, the correlation between the miR-504/FZD7 ratio and ME signature genes was statistically estimated. We observed remarkably lower miR-504/FZD7 ratios in the ME subtype compared with that in PN subtype (*P* < 0.001, Fig. 7a), and low-ratio tumors were significantly enriched in the ME group (P < 0.001, chi-square test, Fig. 7b). In addition, the ME and PN signature genes exhibited distinctly different expression patterns corresponding to the miR-504/FZD7 ratio (Fig. 7c), and the ratio correlated negatively with ME score (R = − 0.580, *P* < 0.001, Pearson correlation test, Fig. 7d) as well as several well-known ME markers, i.e., CD44, YKL-40 (chitinase 3–like 1), vimentin, and SNAI2 (Additional file 11: Figure S5b) in patients with GBM. PCA based on the Verhaak GBM ME and hallmark EMT gene sets showed distinct distribution patterns between GBM in the high- and low-ratio groups, indicating different ME marker and EMT marker expression profiles among the two groups (Fig. 7e). Interestingly, similar results were observed from PCA based on the stem cell proliferation and Wnt signal pathway–related gene sets (Additional file 10: Figure S4c, S4d), confirming the molecular biological experimental findings above. We attempted to illustrate the potential biological functions and signaling pathways related to the miR-504/FZD7 ratio in GBM by computationally analyzing the whole-genome expression profile from TCGA dataset. Genes that were differentially upregulated in low miR-504/FZD7 ratio cases and that correlated negatively with the miR-504/FZD7 ratio were respectively summarized and subsequently submitted to GO analyses. The results indicated that miR-504/FZD7 ratio–associated genes were most involved in ME phenotype–related processes, including cell migration, cell adhesion, collagen fibril organization, and actin cytoskeleton reorganization (Fig. 7f, g). Consistently, GSEA demonstrated that a low miR-504/FZD7 ratio was significantly related to the regulation of cell adhesion and EMT (Fig. 7h). Moreover, GSVA confirmed the significant correlation between the miR-504/FZD7 ratio and cell adhesion, ME–epithelial transition, and stem cell population maintenance (Fig. 7i). Altogether, these data implicate the miR-504/FZD7 ratio as a novel biomarker of the ME subtype and that it is closely related with the ME phenotype as well as EMT in GBM.

### The miR-504/FZD7 ratio was a prognostic and chemotherapeutic and radiotherapeutic indicator for patients with GBM

**Fig. 8:**
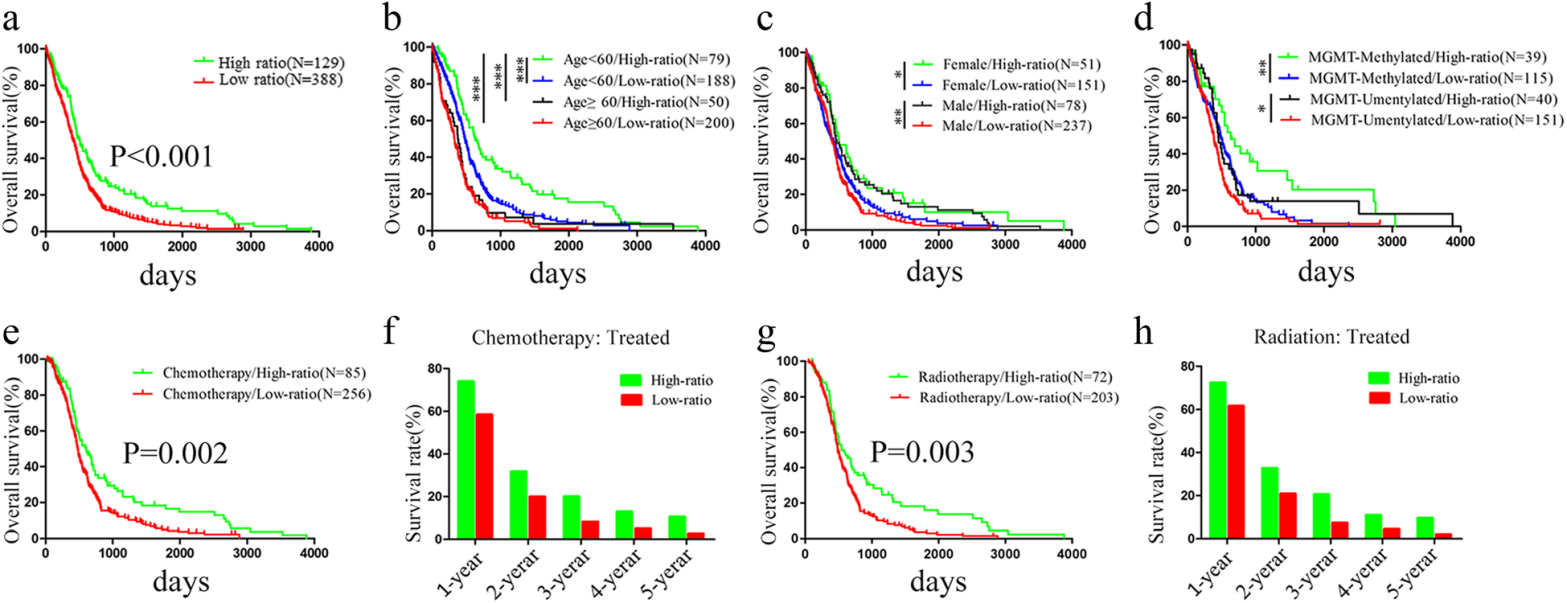
The miR-504/FZD7 ratio is a prognostic indicator in GBM. **a** Kaplan–Meier assessment of the association between the miR-504/FZD7 ratio and overall survival of patients with GBM in TCGA. **b**–**d** Prognostic significance of the miR-504/FZD7 ratio in cohorts stratified by age (**b**), sex (**c**), and MGMT promoter status (**d**). **e** Kaplan–Meier analysis of survival of chemotherapy-treated patients with GBM based on the miR-504/FZD7 ratio. **f** Comparison of 1–5-year survival rates between high and low miR-504/FZD7 ratio groups in chemotherapy-treated patients with GBM. **g** Kaplan–Meier analysis of overall survival according to the miR-504/FZD7 ratio of standard radiotherapy–treated patients with GBM from TCGA. **h** Comparison of 1–5-year survival rates between high and low miR-504/FZD7 ratio groups in standard radiotherapy–treated patients with GBM. *P < 0.05, **P < 0.01, ***P < 0.001

ME subtype GBM has more aggressive phenotypes, leading to therapeutic failure and poor prognosis. Therefore, after identifying the miR-504/FZD7 ratio as a novel marker of ME subtype GBM, we explored application of the ratio for predicting the prognosis and chemotherapeutic and radiotherapeutic sensitivity of patients with GBM. To evaluate the prognostic value of the ratio, we first performed log-rank testing and Kaplan–Meier analysis to statistically assess the association between the ratio and overall survival based on TCGA dataset. Patients with GBM with low miR-504/FZD7 ratios had significantly shorter overall survival compared to patients with high ratios (*P* < 0.001, log rank test, Fig. 8a). Additionally, we performed univariate and multivariate Cox regression analysis and determined that a low miR-504/FZD7 ratio was an independent risk factor of poor prognosis for patients with GBM (Table 1, *P* = 0.024, hazard ratio [HR] = 1.542, multivariate Cox regression analysis). Furthermore, we classified patients by age, sex, MGMT (O-6-methylguanine-DNA methyltransferase) promoter methylation status and IDH1 (isocitrate dehydrogenase [NADP(+)] 1, cytosolic) mutation to estimate the prognostic value of the miR-504/FZD7 ratio in stratified GBM cohorts based on the Kaplan–Meier curves. First, in the younger age group (< 60 years), patients with low miR-504/FZD7 ratios had significantly shorter overall survival than patients with high ratios (Fig. 8b). Impressively, the combination of younger age and high miR-504/FZD7 ratio stratified the patients into a subgroup with remarkably longer survival (Fig. 8b). Second, in both female and male groups, patients with low miR-504/FZD7 ratios had poor survival (Fig. 8c). Third, the analysis combined with MGMT promoter status demonstrated that low miR-504/FZD7 ratios correlated significantly with shorter survival in both methylated and unmethylated MGMT groups (Fig. 8d). Similarly, patients with MGMT promoter methylation and high miR-504/FZD7 ratios had significantly better prognosis than patients who did not (Fig. 8d). Lastly, there was no prognostic significance for the miR-504/FZD7 ratio in the relevant patient subgroups according to IDH1 mutation–based stratification (data not shown). Moreover, we tested the role of the miR-504/FZD7 ratio for predicting the chemotherapeutic and radiotherapeutic responses of the patients with GBM. TCGA data on patients with GBM who underwent postoperative chemotherapy (*n* = 341) and radiotherapy (*n* = 275) were collected and subsequently submitted to survival analyses. Low miR-504/FZD7 ratios were closely correlated with survival disadvantage in both chemotherapy-treated (Fig. 8e, f) and radiotherapy-treated patients (Fig. 8g, h), indicating that a low miR-504/FZD7 ratio is an indicator of chemotherapeutic and radiotherapeutic resistance in patients with GBM. Collectively, these results demonstrate that the miR-504/FZD7 ratio is a predictive marker of prognosis and an indicator of chemotherapeutic and radiotherapeutic response in GBM.

**Table 1:** Univariate and multivariate Cox regression analysis of overall survival in 517 patients with GBM

Variable	Univariate regression		Multivariate regression	
	HR (95%CI)	*P*-value	HR (95%CI)	P-value
Age				
(≥60 vs. < 60 years)	1.794 (1.474–2.183)	< 0.001	1.429 (1.047–1.952)	0.025
Sex				
(Female vs. male)	0.879 (0.726–1.064)			
KPS				
(< 80 vs. ≥80)	2.152 (1.554–2.979)	< 0.001	1.387 (0.968–1.989)	0.075
MGMT promoter status				
(Methylated vs. unmethylated)	0.742 (0.585–0.941)	0.013	0.896 (0.659–1.220)	0.487
Chemotherapy				
(Yes vs. no)	0.434 (0.337–0.557)	< 0.001	0.453 (0.277–0.739)	0.002
Radiotherapy				
(Yes vs. no)	0.571 (0.466–0.701)	< 0.001	0.653 (0.437–0.975)	0.037
Subtype				
ME vs. non-ME	1.269 (1.034–1.558)	0.016	1.323 (0.960–1.822)	0.087
miR-504/FZD7 ratio				
(Low vs. high)	1.511 (1.239–1.844)	< 0.001	1.542 (1.059–2.245)	0.024

*95% CI* 95% confidence interval, *KPS* Karnofsky performance status

## Discussion

Based on integrated transcriptomic and genomic analyses, Verhaak et al. classified GBM into four molecular subtypes: ME, PN, CL, and NE, which have different biological behaviors and distinct markers. Among them, ME subtype GBM has more aggressive properties, such as radioresistance and chemoresistance, increased invasiveness, and reduced cell stiffness, and leading to therapeutic failure and poor prognosis. miRNAs have been widely identified to play crucial roles in regulating ME phenotype transition in GBM. Yang et al. reported remarkably decreased expression of miR-181d in ME subtype GBM compared with PN tumors, in both TCGA and CGGA (Chinese Glioma Genome Atlas) cohorts, and attenuated ME phenotype GBM by repressing nuclear factor kappa B (NFκB) transcriptional activity via direct targeting of MALT1 (MALT1 paracaspase) [28]. Wu et al. found that the miR-155HG–miR-155 axis plays a critical role in ME transition progression by regulating PCDH9 (protocadherin 9) and PCDH7, which play a pivotal role in glioma by suppressing the Wnt–β-catenin pathway, and serves as a prognostic factor of survival in GBM [29]. Here, we found that miR-504 downregulation correlated with ME subtype GBM and many ME transition–related biological processes (cell adhesion, angiogenesis, cell matrix adhesion).

Recently, investigations have implicated the tumor-suppressive role of miR-504 in human cancers, providing evidence that this miRNA can repress cell proliferation and invasion in both hypopharyngeal cell carcinoma and hepatocellular carcinoma (HCC) [30, 31]. Similarly, miR-504 is downregulated in non–small cell lung cancer tissue and inhibits cell proliferation, invasion, and EMT by targeting LOXL2 (lysyl oxidase–like 2) [32]. Consistent with these findings, we have previously shown that miR-504 is downregulated and acts as a tumor suppressor in GBM [14, 20, 21, 33]. Moreover, among these studies, integrated analysis of the correlation between miRNA and mRNA expression has indicated that miR-504 expression correlates with ME markers in GBM tissue, including vimentin and YKL-40 [21]. Here, we found that miR-504 overexpression suppressed the migration and invasive capability of GBM cells, and that inhibiting miR-504 expression had the opposite effect. We also observed that miR-504 suppressed EMT, which plays key roles in promoting aggressive behaviors and is characterized by the loss of epithelial markers (e.g., E-cadherin) and gain of ME markers (e.g., N-cadherin, vimentin, CD44). The existence of GSCs, which are characterized by self-renewal ability and the generation of larger tumor bulk, has been associated with EMT and ME subtype transition [34]. In the present study, overexpression of miR-504 attenuated the stemness activity of GSCs by downregulating the expression of the stem cell markers CD133, nestin, SOX2, and KLF4. These results indicate that miR-504 suppresses ME phenotype GBM differently, i.e., by inhibiting EMT and reducing GSC stemness activity.

FZD7, widely known as the most common reporter of Wnt, has been recognized as a target for cancer therapy, as it can play an important role in controlling endothelial cell proliferation by inhibiting the Wnt–β-catenin signaling regulators [35]. FZD7 is upregulated in multiple solid cancers and is involved in cancer development and progression. Merle and colleagues found high FZD7 expression in HCC tissues and cell lines, and that it correlated with β-catenin accumulation in HCC tumors [36]. Qiu et al. reported FZD7 overexpression in glioma, leading to increased cell proliferation by upregulating tafazzin (TAZ), and that high FZD7 expression predicted poor overall survival [37]. To date, several miRNAs, such as miR-485-5p [38], miR-488 [39], miR-144-3p [27], and miR-27b [40] inhibit cancer progression by targeting FZD7. In a more recent study, Chen et al. observed that FZD7 was targeted by miR-638 and upregulated by hsa_circ_0000177, and contributed to malignant behaviors in glioma [26]. In the present study, we show that FZD7 was a direct target of miR-504. Overexpression of miR-504 decreased FZD7 mRNA and protein expression levels. Moreover, miR-504 expression correlated negatively with FZD7 expression in GBM tissue.

The Wnt–β-catenin signaling pathway plays an important role in tumor development and promotes tumor invasiveness by inducing EMT and cancer cell stemness. In several types of cancer, β-catenin is sequestered by E-cadherin in the cytoplasm, with β-catenin nuclear translocation following the downregulation of E-cadherin correlating directly with acquisition of the ME phenotype [41, 42]. On the other hand, the activated Wnt–β-catenin pathway triggers a set of EMT activators, including TWIST1 (twist family bHLH transcription factor 1), SNAI1, and SNAI2. Moreover, a number of Wnt target genes, such as SOX2, CD44, and LGR5 (leucine-rich repeat–containing G protein–coupled receptor 5), have been widely proven to maintain the stemness of GSCs [43, 44]. Accumulated evidence shows that miRNAs target key components of the Wnt–β-catenin pathway directly and regulate the biological function of cancer cells. In colon cancer cells, miR-147 inhibits stem cell markers and EMT-related protein expression via the Wnt–β-catenin signaling pathway [45]. Jin et al. found that Let-7 inhibits self-renewal of hepatocellular cancer stem-like cells by regulating EMT and the Wnt signaling pathway [46]. FZD7, one of the frizzled family of Wnt ligand receptors, functions as a positive regulator of the Wnt signaling pathway. Here, we demonstrate that FZD7 is a direct target of miR-504. Further, we found that overexpression of miR-504 blocked Wnt signaling activation, reduced β-catenin and p-GSK3β expression, decreased β-catenin nuclear accumulation, and inhibited the expression of the downstream target genes CD44 and c-MYC. However, rescue experiments showed that overexpressing FZD7 partially reversed Wnt signaling pathway activity and ME behaviors and signatures, which miR-504 had suppressed, indicating that miR-504–FZD7–Wnt–β-catenin play an important role in the molecular pathogenesis of GBM.

Moreover, accumulating evidence indicates that combining multiple molecular markers can provide superior prognostic value compared with individual marker–based analysis [47, 48]. Shi et al. reported that miR-663 suppresses the oncogenic function of CXCR4 (C-X-C motif chemokine receptor 4), and composed a prognostic biomarker set for GBM [49]. Mlcochova et al. created an EMT-associated miRNA/mRNA signature linked to metastasis and prognosis in clear cell renal cell carcinoma [50]. Our results suggest that the miR-504/FZD7 ratio could serve as a novel biomarker for ME subtype GBM and is an independent prognostic predictor in GBM. The ratio also showed significant prognostic value in GBM cohorts stratified by age, sex, and MGMT promoter methylation status. Furthermore, our results indicate that the miR-504/FZD7 ratio correlates closely with ME signatures and EMT, and can be used as a significant indicator of chemotherapeutic and radiotherapeutic response in GBM.

## Conclusions

miR-504 suppresses the ME phenotype and EMT in GBM by directly inhibiting the FZD7-mediated Wnt–β-catenin signaling pathway. Importantly, the miR-504/FZD7 ratio is a novel biomarker of ME subtype GBM and an indicator of survival and treatment outcome in patients with GBM. Our study might provide novel insights into understanding the molecular pathogenesis of GBM and facilitate the development of miRNA-based GBM therapy.

## Additional files

Additional file 1: **Table S1.** The annotated gene sets involved in this study. (XLSX 31 kb)

Additional file 2: Information on the lentivirus and plasmid sequences used in this study. (DOCX 272 kb)

Additional file 3: **Table S2.** Primer information. (DOCX 17 kb)

Additional file 4: **Table S3.** miR-504–related genes. (XLSX 17 kb)

Additional file 5: **Figure S1.** (a–d) The prognostic value of miR-504 in the four molecular subtypes of GBM. (TIF 1955 kb)

Additional file 6: **Table S4.** List of top 200 significant differentially upregulated genes in miR-504 low-expression tumors. (XLSX 14 kb)

Additional file 7: **Figure S2.** (a, b) Tube formation assay detection of the effect of miR-504 on angiogenesis.**P < 0.01,***P < 0.001 (TIF 4824 kb)

Additional file 8: miR-504 inhibits invasiveness of GBM cells in vivo. a. H&E staining of sections at tumor margins in intracranial miR-NC-U87 and miR-504-U87 xenografts. b. The size of intracranial miR-NC-U87 and miR-504-U87 xenografts were measured. c. Survival analysis for animals implanted with miR-NC-U87 or miR-504-U87 cells. d. IHC for β-catenin and vimentin in sections from indicated xenografts. (TIF 3133 kb)

Additional file 9: **Table S5.** Candidate genes predicted by TargetScan, miRDB, and PITA. (XLSX 38 kb)

Additional file 10: **Figure S4.** (a) Representative immunohistochemical staining of FZD7 in GBM specimens stratified by miR-504 expression. (b) Pearson chi-square analysis of the correlation of miR-504 and FZD7 protein expression.*P < 0.05. (TIF 2559 kb)

Additional file 11: **Figure S5.** The miR-504/FZD7 ratio correlated with the ME signature genes. (a) Distribution of samples based on whole-genome expression data from TCGA. (b) The correlation between the miR-504/FZD7 ratio and ME markers (CD44, YKL-40, vimentin, SNAI2). (c, d) PCA of TCGA data showing different distributions in the stem cell proliferation and canonical Wnt–β-catenin pathway–related genes between the high and low miR-504/FZD7 ratio groups. (TIF 864 kb)

## Data Availability

The datasets generated and/or analyzed during the current study are available in TCGA (http://cancergenome.nih.gov). The associated GO gene sets were obtained from MSigDB (http://software.broadinstitute.org/gsea/msigdb).

## Abbreviations

CGGA: Chinese Glioma Genome Altas
CL: Classical
EMT: Epithelial-Mesenchymal Transition
FBS: fetal bovine serum
FDR: False discovery rate
FZD7: Frizzeled-7
GBM: Glioblastoma
GFP: Green fluorescent protein
GO: Gene Ontology
GSCs: Glioma Stem Cells
GSEA: Gene Set Enrichment Analysis
GSVA: Gene Set Variation Analysis
KPS: Karnofsky Performance Status
ME: Mesenchymal
miRNA: MicroRNA
NBT: Normal brain tissue
NE: Neural
NES: Normalized enrichment score
PCA: Principal components analysis
PN: Proneural
qRT-PCR: Real-time Quantitative Polymerase Chain Reaction
SFE: Sphere formation efficiency
TCGA: The Cancer Genome Atlas;
