# Supplementary material for: miR-504 suppresses mesenchymal phenotype of glioblastoma by directly targeting the FZD7-mediated Wnt–β-catenin pathway
Source: J Exp Clin Cancer Res. 2019 Aug 16;38:358. doi: 10.1186/s13046-019-1370-1 (PMC6697940; doi:10.1186/s13046-019-1370-1)

**1.Information of lentiviral vector for miR-504 overexpression**

**pri-miR-504:** CGAAGCTTGGGCTGCAGGTCGACTCTAGAGGATCCCCGGGTACCGGAGTGGAATAACTCAAAAGGAAAAGGTAACTCAACGAGCCATCCATTTCCCACATAGAATCACAACAGAAGACAGAAAATATGGAGTGGAATGTTGAATCAGCTGCTGTTGGGAGACCCTGGTCTGCACTCTATCTGTATTCTTACTGAAGGGAGTGCAGGGCAGGGTTTCCCATACAGAGGGCACAGACTACCACCGTATGGCAAATGTTGACTGGAACTCAACTTCAAAGCCATTTCACCTGGAATCTTACCCCACCACCCCCTAGCCTGTGGAATGTGTGTCAGTTAGGGTGTGGAAAGTCCCCAGGCTCCCCAGCAGGCAGAAGTATGCAAAGCATG

Vector：GV369

**
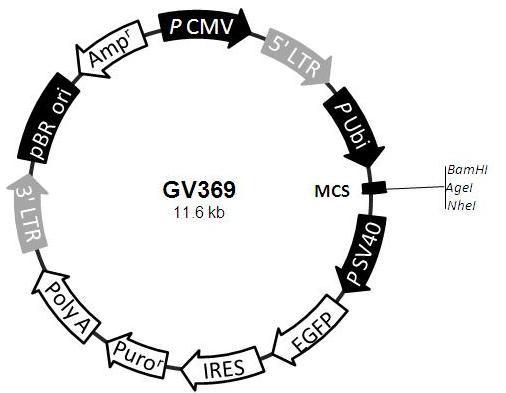
**

**2 Information of lentiviral vector for miR-504 inhibition**

**miR-504inhibiton** AaTTACAAAAACAAATTACAAAAATTCAAAATTTTCGGGTTTATTACAGGGACAGCAGAGATCCAGTTTGGTTAGTACCGGGCCCGCTCTAGACTCGAGATATTTGCATGTCGCTATGTGTTCTGGGAAATCACCATAAACGTGAAATGTCTTTGGATTTGGGAATCTTATAAGTTCTGTATGAGACCACTCACCGGGATAGAGTGCAGACCAGGGTCTTTTTTGAATTCGGATCCATTAGGCGGCCGCGTGGATAACCGTATTACCGCCATGCATTAGTTATTAATAGTAATCAATTACGGGGTCATTAGTTCATAGCCCATATATGGAGTTCCGCGTTACATAACTTACGGTAAATGGCCCGCCTGGCTGACCGCCCAACGACCCCCGCCCATTGACGTCAATAATGACGTATGTTCCCATAGTAACGCCAATAGGGACTTTCCATTGACGTCAATGGGTGGAGTATTTACGGTAAACTGCCCACTTGGCAGTACATCAAGTGTATCATATGCCAAGTACGCCCCCTATTGACGTCAATGACGGTAAATGGCCCGCCTGGCATTATGCCCAGTACATGACCTTATGGGACTTTCCTACTTGGCAGTACATCTACGTATTAGTCATCGCTATTACCATGGTGATGCGGTTTTGGCAGTACATCAATGGGCGTGGATAGCGGTTTGACTCACGGGGATTTCCAAGTCTCCACCCCATTGACGTCAATGGGAGTTTGTTTTGGCACCAAAATCAACGGGACTTTCCAAAATGTCGTAACAACTCCGCCCCATTGACGCAAATGGGCGGTAGGCGTGTACGGTGGGAGGTCTATATAAGCAGAGCTGGTTTAGTGAACCGTCAGATCCGCTAGCGCTACCGGACGCCACCATGGTGAGCAAGGGCGAGGAGCTGTTCACCGGGGTGTGCCCATCCTGGTCGAGCTGGACGGCGACGTAAACGGCCACAAGTTCAGCGTGTCCGGCGGAGGGCGAGGGCGATGCCACCTACGGCAAG

**(yellow: target sequence)**

Vector：GV159


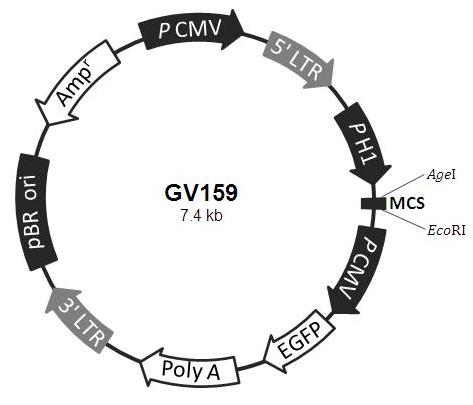


**3. Information of lentiviral vector for FZD7 overexpression**

**FZD7**

CTTTTTTGTTAGACGAAGCTTGGGCTGCAGGTCGACTCTAGAGGATCCCCGGGTACCGGTCGCCACCATGCGGGACCCCGGCGCGGCCGCTCCGCTTTCGTCCCTGGGCCTCTGTGCCCTGGTGCTGGCGCTGCTGGGCGCACTGTCCGCGGGCGCCGGGGCGCAGCCGTACCACGGAGAGAAGGGCATCTCCGTGCCGGACCACGGCTTCTGCCAGCCCATCTCCATCCCGCTGTGCACGGACATCGCCTACAACCAGACCATCCTGCCCAACCTGCTGGGCCACACGAACCAAGAGGACGCGGGCCTCGAGGTGCACCAGTTCTACCCGCTGGTGAAGGTGCAGTGTTCTCCCGAACTCCGCTTTTTCTTATGCTCCATGTATGCGCCCGTGTGCACCGTGCTCGATCAGGCCATCCCGCCGTGTCGTTCTCTGTGCGAGCGCGCCCGCCAGGGCTGCGAGGCGCTCATGAACAAGTTCGGCTTCCAGTGGCCCGAGCGGCTGCGCTGCGAGAACTTCCCGGTGCACGGTGCGGGCGAGATCTGCGTGGGCCAGAACACGTCGGACGGCTCCGGGGGCCCAGGCGGCGGCCCCACTGCCTACCCTACCGCGCCCTACCTGCCGGACCTGCCCTTCACCGCGCTGCCCCCGGGGGCCTCAGATGGCAGGGGGCGTCCCGCCTTCCCCTTCTCATGCCCCCGTCAGCTCAAGGTGCCCCCGTACCTGGGCTACCGCTTCCTGGGTGAGCGCGATTGTGGCGCCCCGTGCGAACCGGGCCGTGCCAACGGCCTGATGTACTTTAAGGAGGAGGAGAGGCGCTTCGCCCGCCTCTGGGTGGGCGTGTGGTCCGTGCTGTGCTGCGCCTCGACGCTCTTTACCGTTCTCACCTACCTGGTGGACATGCGGCGCTTCAGCTACCCAGAGCGGCCCATCATCTTCCTGTCGGGCTGCTACTTCATGGTGGCCGTGGCGCACGTGGCCGGCTTCCTTCTAGAGGACCGCGCCGTGTGCGTGGAGCGCTTCTCGGACGATGGCTACCGCACGGTGGCGCAGGGCACCAAGAAGGAGGGCTGCACCATCCTCTTCATGGTGCTCTACTTCTTCGGCATGGCCAGCTCCATCTGGTGGGTCATTCTGTCTCTCACTTGGTTCCTGGCGGCCGGCATGAAGTGGGGCCACGAGGCCATCGAGGCCAACTCGCAGTACTTCCACCTGGCCGCGTGGGCCGTGCCCGCCGTCAAGACCATCACTATCCTGGCCATGGGCCAGGTAGACGGGGACCTGCTGAGCGGGGTGTGCTACGTTGGCCTCTCCAGTGTGGACGCGCTGCGGGGCTTCGTGCTGGCGCCTCTGTTCGTCTACCTCTTCATAGGCACGTCCTTCTTGCTGGCCGGCTTCGTGTCCCTCTTCCGTATCCGCACCATCATGAAACACGACGGCACCAAGACCGAGAAGCTGGAGAAGCTCATGGTGCGCATCGGCGTCTTCAGCGTGCTCTACACAGTGCCCGCCACCATCGTCCTGGCCTGCTACTTCTACGAGCAGGCCTTCCGCGAGCACTGGGAGCGCACCTGGCTCCTGCAGACGTGCAAGAGCTATGCCGTGCCCTGCCCGCCCGGCCACTTCCCGCCCATGAGCCCCGACTTCACCGTCTTCATGATCAAGTACCTGATGACCATGATCGTCGGCATCACCACTGGCTTCTGGATCTGGTCGGGCAAGACCCTGCAGTCGTGGCGCCGCTTCTACCACAGACTTAGCCACAGCAGCAAGGGGGAGACTGCGGTAGGTATGGACTACAAGGATGACGATGACAAGGATTACAAAGACGACGATGATAAGGACTATAAGGATGATGACGACAAATGAGCTAGCCTGTGGAATGTGTGTCAGTTAGGGTGTGGAAAGTCCCCAGGCTCCCCAGCAGGCAGAA

Vector：GV320

**
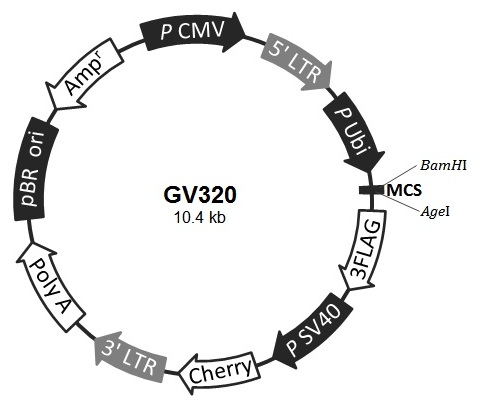
**

**4.FZD7 3’UTR wildtype**

CTACCGGAAACTCGACGCAAGAAAATCAGAGAGATCCTCATAAAGGCCAAGAAGGGCGGAAAGTCCAAATTGCTCGAGTGATGAAAGCTGCGCACTAGTGAGGGAGCTCATAGGCCGGCATAGACGCGTGCCCCGGCCCCTCCCCACCTTTCCCACCCCAGCCCTCTTGCAAGAGGAGAGGCACGGTAGGGAAAAGAACTGCTGGGTGGGGGCCTGTTTCTGTAACTTTCTCCCCCTCTACTGAGAAGTGACCTGGAAGTGAGAAGTTCTTTGCAGATTTGGGGCGAGGGGTGATTTGGAAAAGAAGACCTGGGTGGAAAGCGGTTTGGATGAAAAGATTTCAGGCAAAGACTTGCAGGAAGATGATGATAACGGCGATGTGAATCGTCAAAGGTACGGGCCAGCTTGTGCCTAATAGAAGGTTGAGACCAGCAGAGACTGCTGTGAGTTTCTCCCGGCTCCGAGGCTGAACGGGGACTGTGAGCGATCCCCCTGCTGCAGGGCGAGTGGCCTGTCCAGACCCCTGTGAGGCCCCGGGAAAGGTACAGCCCTGTCTGCGGTGGCTGCTTTGTTGGAAAGAGGGAGGGCCTCCTGCGGTGTGCTTGTCAAGCAGTGGTCAAACCATAATCTCTTTTCACTGGGGCCAAACTGGAGCCCAGATGGGTTAATTTCCAGGGTCAGACATTACGGTCTCTCCTCCCCTGCCCCCTCCCGCCTGTTTTTCCTCCCGTACTGCTTTCAGGTCTTGTAAAATAAGCATTTGGAAGTCTTGGGAGGCCTGCCTGCTAGAATCCTAATGTGAGGATGCAAAAGAAATGATGATAACATTTTGAGATAAGGCCAAGGAGACGTGGAGTAGGTATTTTTGCTACTTTTTCATTTTCTGGGGAAGGCAGGAGGCAGAAAGACGGGTGTTTTATTTGGTCTAATACCCTGAAAAGAAGTGATGACTTGTTGCTTTTCAAAACAGGAATGCATTTTTCCCCTTGTCTTTGTTGTAAGAGACAAAAGAGGAAACAAAAGTGTCTCCCTGTGGAAAGGCATAACTGTGACGAAAGCAACTTTTATAGGCAAAGCAGCGCAAATCTGAGGTTTCCCGTTGGTTGTTAATTTGGTTGAGATAAACATTCCTTTTTAAGGAAAAGTGAAGAGCAGTGTGCTGTCACACACCGTTAAGCCAGAGGTTCTGACTTCGCTAAAGGAAATGTAAGAGGTTTTGTTGTCTGTTTTAAATAAATTTAATTCGGAACACATGATCCAACAGACTATGTTAAAATATTCAGGGAAATCTCTCCCTTCATTTACTTTTTCTTGCTATAAGCCTATATTTAGGTTTCTTTTCTATTTTTTTCTCCCATTTGGATCCTTTGAGGTAAAAAAACATAATGTCTTCAGCCTCATAATAAAGGAAAGTTAATTAAAAAAAAAGCAAAGAGCCATTTTGTCCTGTTTTCTTGGTTCCATCAATCTGTTTATTAAACATCATCCATATGCTGACCCTGTCTCTGTGTGGTTGGGTTGGGAGGCGATCAGCAGATACCATAGTGAACGAAGAGGAAGGTTTGAACCATGGGCCCCATCTTTAAAGAAAGTCATTAAAAGAAGGTAAACTTCAAAGTGATTCTGGAGTTCTTTGAAATGTGCTGGAAGACTTAAATTTATTAATCTTAAATCATGTACTTTTTTTCTGTAATAGAACTCGGATTCTTTTGCATGATGGGGTAAAGCTTAGCAGAGAATCATGGGAGCTAACCTTTATCCCACCTTTGACACTACCCTCCAATCTTGCAACACTATCCTGTTTCTCAGAACAGTTTTTAAATGCCAATCATAGAGGGTACTGTAAAGTGTACAAGTTACTTTATATATGTAATGTTCACTTGAGTGGAACTGCTTTTTACATTAAAGTTAAAATCGATCTTGTGTTTCTTCAACCTTCAAAACTATCTCATCTGTCAGATTTTTAAAACTCCAACACAGGTTTTGGCATCTTTTGTGCTGTATCTTTTAAGTGCATGTGAAATTTGTAAAATAGAGATAAGTACAGTATGTATATTTTGTAAATCTCCCATTTTTGTAAGAAAATATATATTGTATTTATACATTTTTACTTTGGATTTTTGTTTTGTTGGCTTTAAAGGTCTACCCCACTTTATCACATGTACAGATCACAAATAAATTTTTTTAAATACAAGCTTAATAAAGGATCTTTTATTTTCATTGGATCTGTGTGTTTGGTTTTTTGTATGCGGCCGCTAGCAT

(yellow:miR-504 target sequence)

Vector：H5994


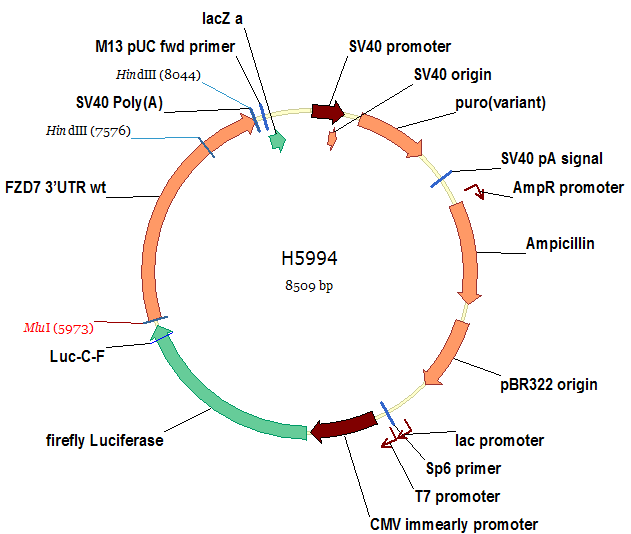


**5.FZD7 3’UTR mutant**

CTACCGGAAACTCGACGCAAGAAAATCAGAGAGATCCTCATAAAGGCCAAGAAGGGCGGAAAGTCCAAATTGCTCGAGTGATGAAAGCTGCGCACTAGTGAGGGAGCTCATAGGCCGGCATAGACGCGTGCCCCGGCCCCTCCCCACCTTTCCCACCCCAGCCCTCTTGCAAGAGGAGAGGCACGGTAGGGAAAAGAACTGCTGGGTGGGGGCCTGTTTCTGTAACTTTCTCCCCCTCTACTGAGAAGTGACCTGGAAGTGAGAAGTTCTTTGCAGATTTGGGGCGAGGGGTGATTTGGAAAAGAAGACCTGGGTGGAAAGCGGTTTGGATGAAAAGATTTCAGGCAAAGACTTGCAGGAAGATGATGATAACGGCGATGTGAATCGTCAAAGGTACGGGCCAGCTTGTGCCTAATAGAAGGTTGAGACCAGCAGAGACTGCTGTGAGTTTCTCCCGGCTCCGAGGCTGAACGGGGACTGTGAGCGATCCCCCTGCTGCAGGGCGAGTGGCCTGTCCAGACCCCTGTGAGGCCCCGGGAAAGGTACAGCCCTGTCTGCGGTGGCTGCTTTGTTGGAAAGAGGGAGGGCCTCCTGCGGTGTGCTTGTCAAGCAGTGGTCAAACCATAATCTCTTTTCACTGGGGCCAAACTGGAGCCCAGATGGGTTAATTTTACATCGCAGACATTACGGTCTCTCCTCCCCTGCCCCCTCCCGCCTGTTTTTCCTCCCGTACTGCTTTCAGGTCTTGTAAAATAAGCATTTGGAAGTCTTGGGAGGCCTGCCTGCTAGAATCCTAATGTGAGGATGCAAAAGAAATGATGATAACATTTTGAGATAAGGCCAAGGAGACGTGGAGTAGGTATTTTTGCTACTTTTTCATTTTCTGGGGAAGGCAGGAGGCAGAAAGACGGGTGTTTTATTTGGTCTAATACCCTGAAAAGAAGTGATGACTTGTTGCTTTTCAAAACAGGAATGCATTTTTCCCCTTGTCTTTGTTGTAAGAGACAAAAGAGGAAACAAAAGTGTCTCCCTGTGGAAAGGCATAACTGTGACGAAAGCAACTTTTATAGGCAAAGCAGCGCAAATCTGAGGTTTCCCGTTGGTTGTTAATTTGGTTGAGATAAACATTCCTTTTTAAGGAAAAGTGAAGAGCAGTGTGCTGTCACACACCGTTAAGCCAGAGGTTCTGACTTCGCTAAAGGAAATGTAAGAGGTTTTGTTGTCTGTTTTAAATAAATTTAATTCGGAACACATGATCCAACAGACTATGTTAAAATATTCAGGGAAATCTCTCCCTTCATTTACTTTTTCTTGCTATAAGCCTATATTTAGGTTTCTTTTCTATTTTTTTCTCCCATTTGGATCCTTTGAGGTAAAAAAACATAATGTCTTCAGCCTCATAATAAAGGAAAGTTAATTAAAAAAAAAGCAAAGAGCCATTTTGTCCTGTTTTCTTGGTTCCATCAATCTGTTTATTAAACATCATCCATATGCTGACCCTGTCTCTGTGTGGTTGGGTTGGGAGGCGATCAGCAGATACCATAGTGAACGAAGAGGAAGGTTTGAACCATGGGCCCCATCTTTAAAGAAAGTCATTAAAAGAAGGTAAACTTCAAAGTGATTCTGGAGTTCTTTGAAATGTGCTGGAAGACTTAAATTTATTAATCTTAAATCATGTACTTTTTTTCTGTAATAGAACTCGGATTCTTTTGCATGATGGGGTAAAGCTTAGCAGAGAATCATGGGAGCTAACCTTTATCCCACCTTTGACACTACCCTCCAATCTTGCAACACTATCCTGTTTCTCAGAACAGTTTTTAAATGCCAATCATAGAGGGTACTGTAAAGTGTACAAGTTACTTTATATATGTAATGTTCACTTGAGTGGAACTGCTTTTTACATTAAAGTTAAAATCGATCTTGTGTTTCTTCAACCTTCAAAACTATCTCATCTGTCAGATTTTTAAAACTCCAACACAGGTTTTGGCATCTTTTGTGCTGTATCTTTTAAGTGCATGTGAAATTTGTAAAATAGAGATAAGTACAGTATGTATATTTTGTAAATCTCCCATTTTTGTAAGAAAATATATATTGTATTTATACATTTTTACTTTGGATTTTTGTTTTGTTGGCTTTAAAGGTCTACCCCACTTTATCACATGTACAGATCACAAATAAATTTTTTTAAATACAAGCTTAATAAAGGATCTTTTATTTTCATTGGATCTGTGTGTTTGGTTTTTTGTATGCGGCCGCTAGCAT

(yellow: mutant site)

Vector：H5995


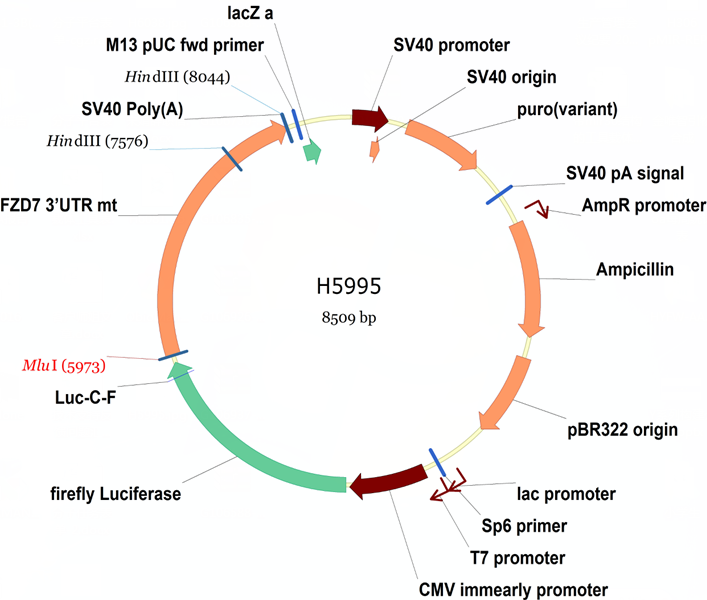

Supplement: Supplementary file 2 — Information on the lentivirus and plasmid sequences used in this study. (DOCX 272 kb) [file 13046_2019_1370_MOESM2_ESM.docx]
