# Supplementary material for: miR-504 suppresses mesenchymal phenotype of glioblastoma by directly targeting the FZD7-mediated Wnt–β-catenin pathway
Source: J Exp Clin Cancer Res. 2019 Aug 16;38:358. doi: 10.1186/s13046-019-1370-1 (PMC6697940; doi:10.1186/s13046-019-1370-1)
Supplement: Supplementary file 3 — Table S2. Primer information. (DOCX 17 kb) [file 13046_2019_1370_MOESM3_ESM.docx]

**Table S2. Information of primers**

| qRT-PCR | |
| --- | --- |
| miR-504 Forward | AGACCCUGGUCUGCACUCUAUC |
| FZD7 Forward | TTATAGGCAAAGCAGCGCAAATC |
| FZD7 Reverse | CCTCTGGCTTAACGGTGTGTGA |
| CD44 Forward | AGAAGGTGTGGGCAGAAGAA |
| CD44 Reverse | AAATGCACCATTTCCTGAGA |
| c-myc Forward | GTCAAGAGGCGAACACACAAC |
| c-myc Reverse | TTGGACGGACAGGATGTATGC |
| GAPDH Forward | GAAGGTGAAGGTCGGAGTC |
| GAPDH Reverse | GAAGATGGTGATGGGATTTC |
