# Supplementary figures and images for: miR-504 suppresses mesenchymal phenotype of glioblastoma by directly targeting the FZD7-mediated Wnt–β-catenin pathway
Source: J Exp Clin Cancer Res. 2019 Aug 16;38:358. doi: 10.1186/s13046-019-1370-1 (PMC6697940; doi:10.1186/s13046-019-1370-1)

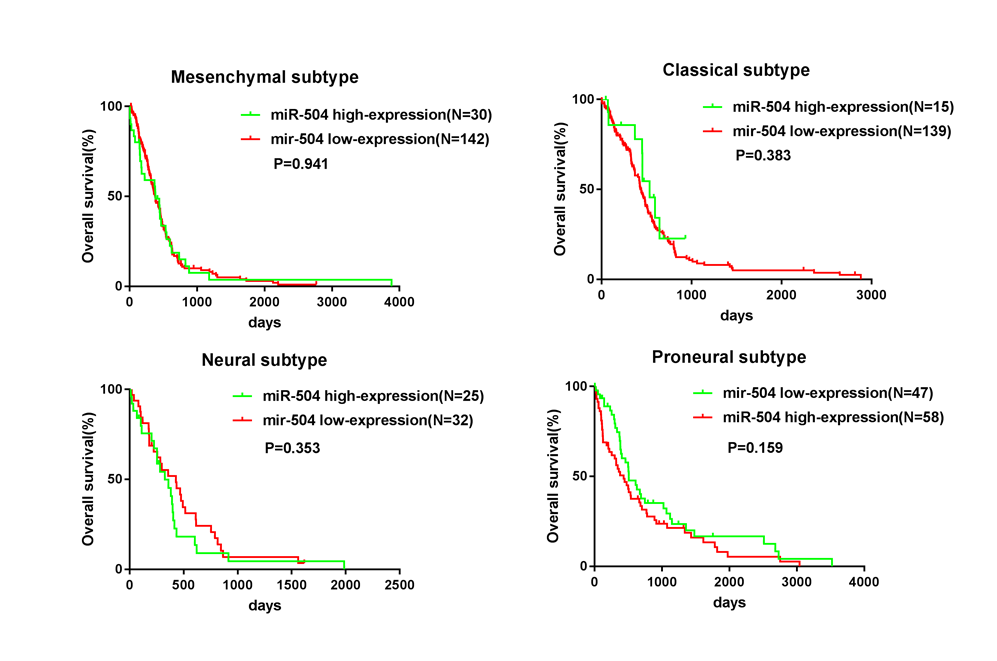

Supplement: Supplementary file 5 — Figure S1. (a–d) The prognostic value of miR-504 in the four molecular subtypes of GBM. (TIF 1955 kb) [file 13046_2019_1370_MOESM5_ESM.tif]

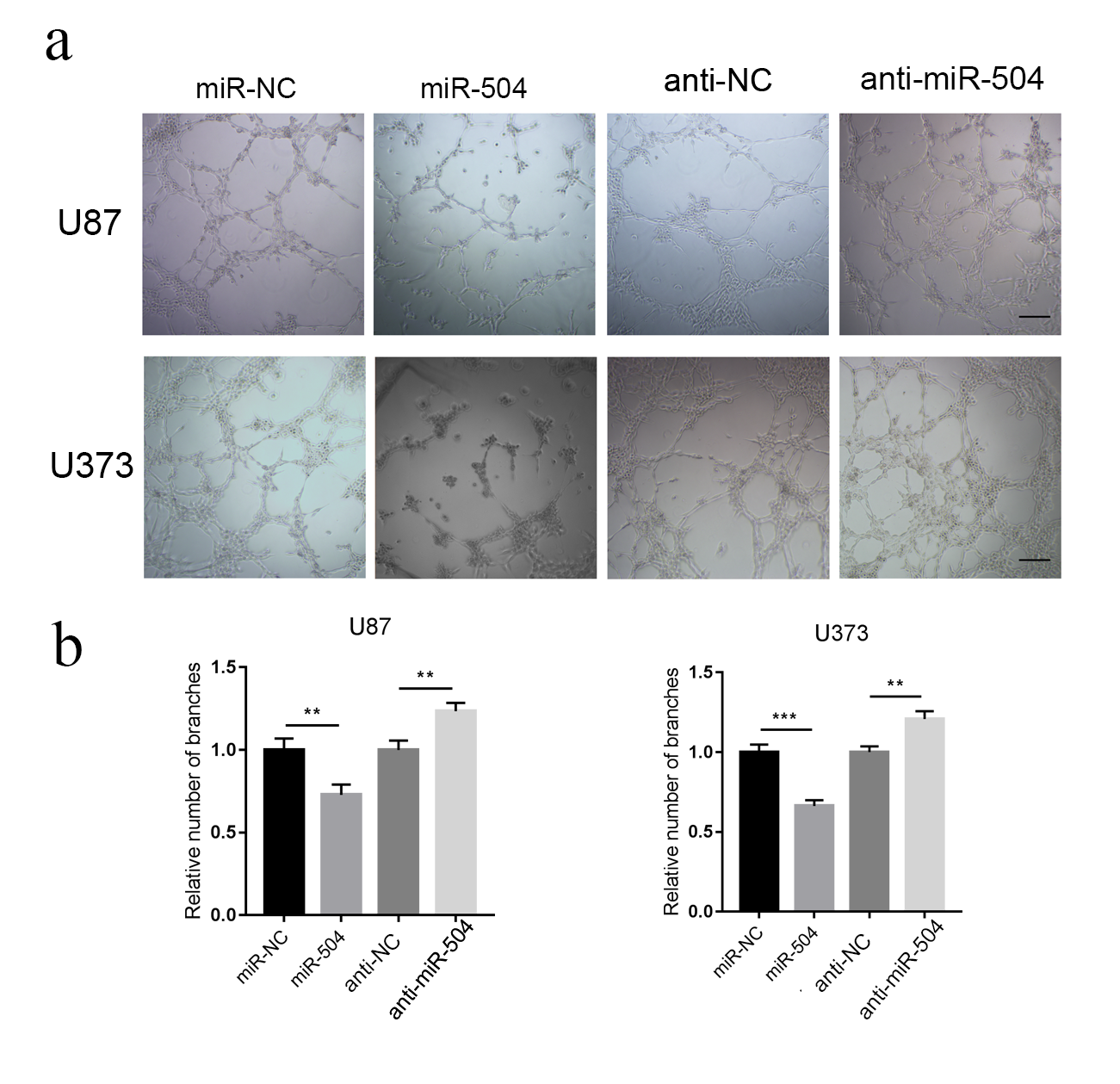

Supplement: Supplementary file 7 — Figure S2. (a, b) Tube formation assay detection of the effect of miR-504 on angiogenesis.**P < 0.01,***P < 0.001 (TIF 4824 kb) [file 13046_2019_1370_MOESM7_ESM.tif]

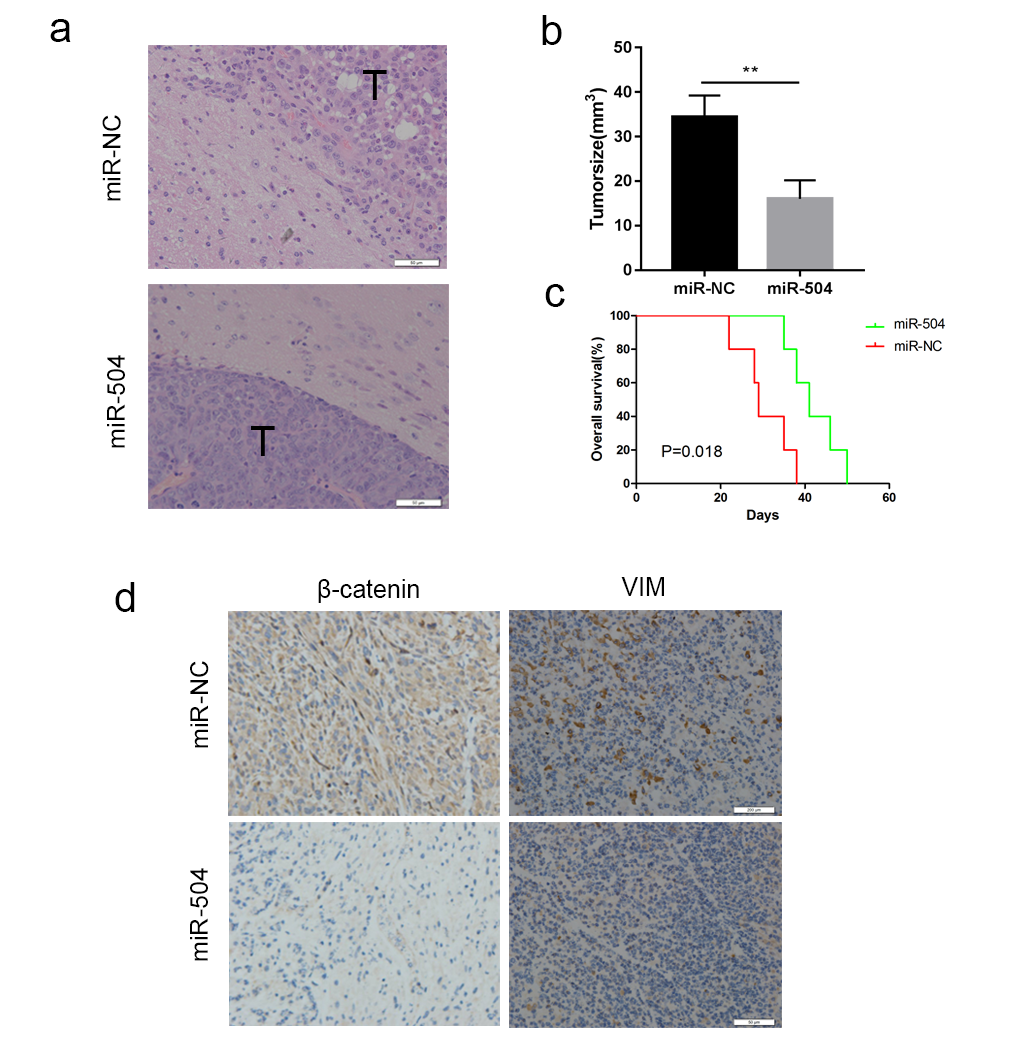

Supplement: Supplementary file 8 — miR-504 inhibits invasiveness of GBM cells in vivo. a. H&E staining of sections at tumor margins in intracranial miR-NC-U87 and miR-504-U87 xenografts. b. The size of intracranial miR-NC-U87 and miR-504-U87 xenografts were measured. c. Survival analysis for animals implanted with miR-NC-U87 or miR-504-U87 cells. d. IHC for β-catenin and vimentin in sections from indicated xenografts. (TIF 3133 kb) [file 13046_2019_1370_MOESM8_ESM.tif]

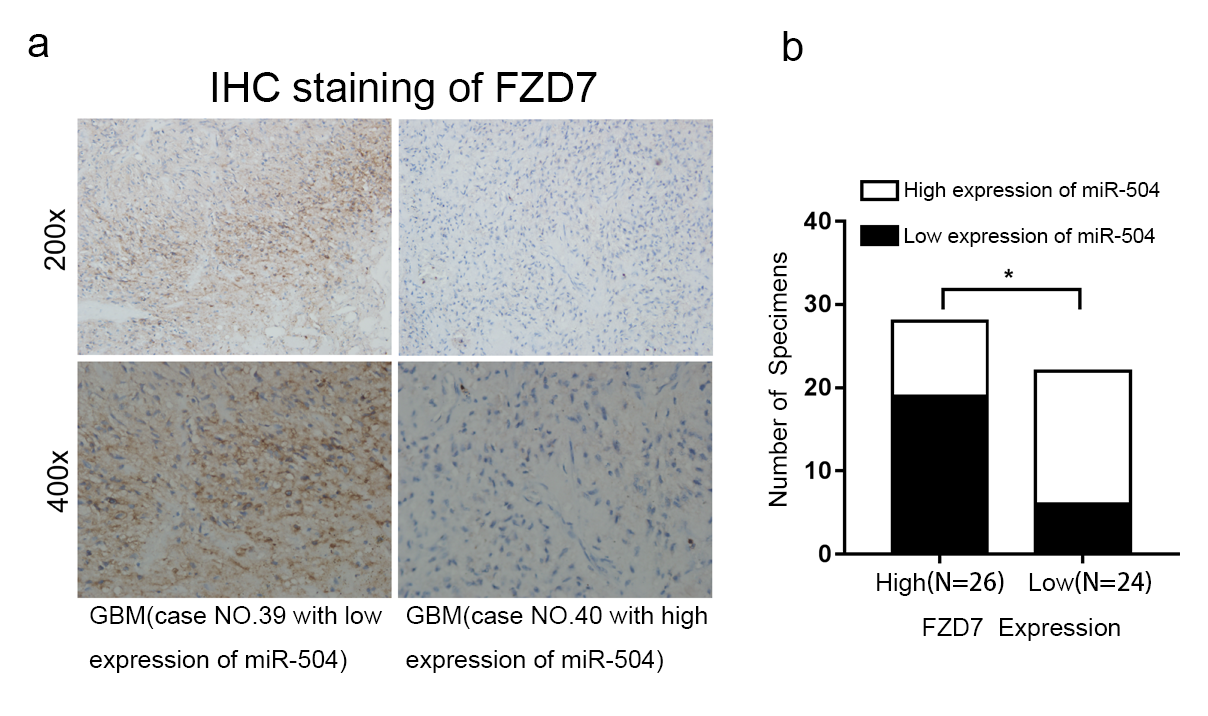

Supplement: Supplementary file 10 — Figure S4. (a) Representative immunohistochemical staining of FZD7 in GBM specimens stratified by miR-504 expression. (b) Pearson chi-square analysis of the correlation of miR-504 and FZD7 protein expression.*P < 0.05. (TIF 2559 kb) [file 13046_2019_1370_MOESM10_ESM.tif]

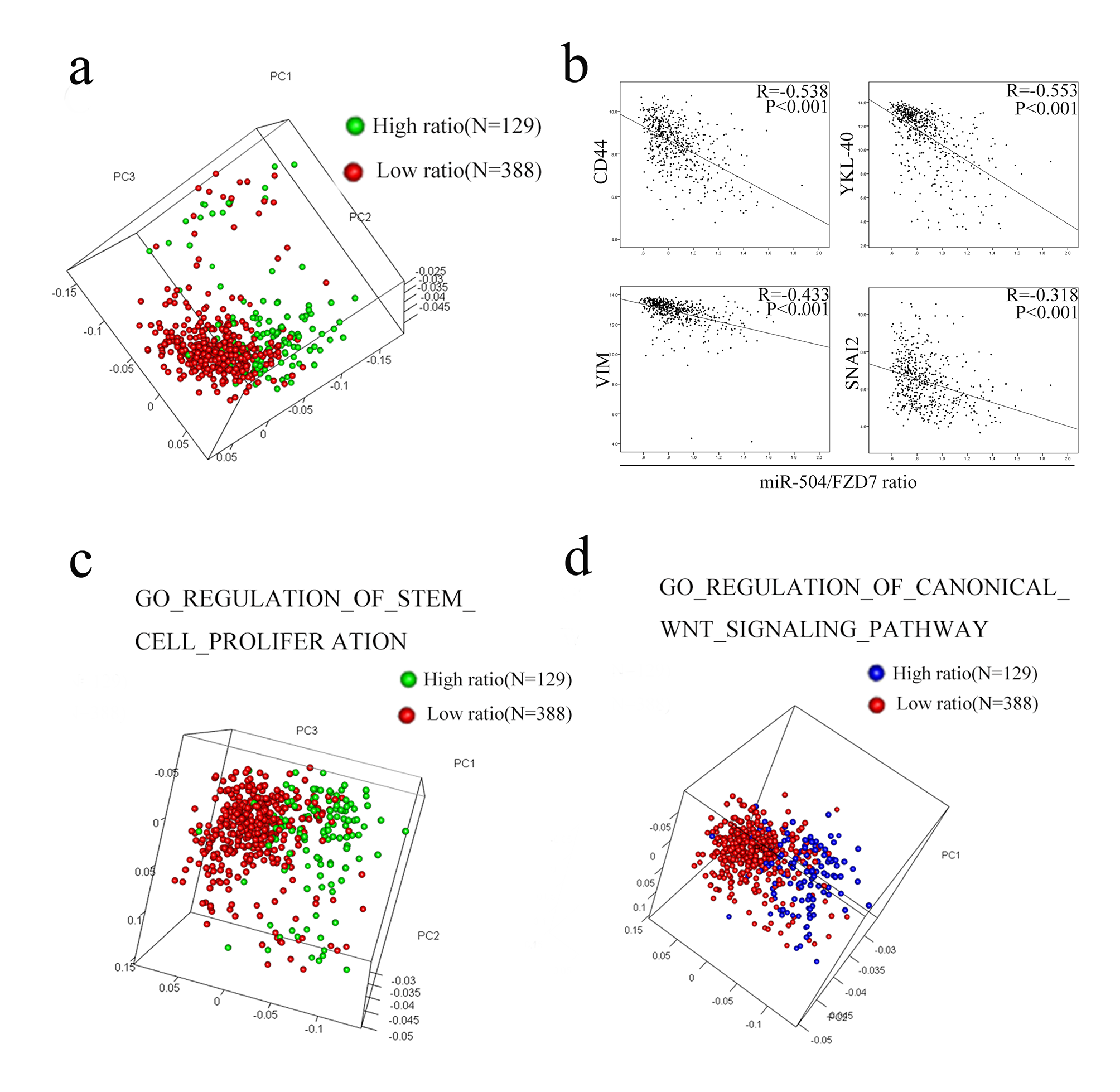

Supplement: Supplementary file 11 — Figure S5. The miR-504/FZD7 ratio correlated with the ME signature genes. (a) Distribution of samples based on whole-genome expression data from TCGA. (b) The correlation between the miR-504/FZD7 ratio and ME markers (CD44, YKL-40, vimentin, SNAI2). (c, d) PCA of TCGA data showing different distributions in the stem cell proliferation and canonical Wnt–β-catenin pathway–related genes between the high and low miR-504/FZD7 ratio groups. (TIF 864 kb) [file 13046_2019_1370_MOESM11_ESM.tif]
